# Beyond Invariable Sites: Using Evolutionary Stasis to Map Multilayered Constraints on the Evolution of Viral and Mammalian Genomes

**DOI:** 10.1093/gbe/evag184

**Published:** 2026-07-28

**Authors:** Sergei L Kosakovsky Pond, Hannah Verdonk, Steven Weaver, Gallean Brown, Danielle Callan, Anton Nekrutenko, Darren P Martin

**Affiliations:** Department of Biology, Temple University, Philadelphia, PA 19122-6078, USA; Department of Biology, Temple University, Philadelphia, PA 19122-6078, USA; Department of Biology, Temple University, Philadelphia, PA 19122-6078, USA; Department of Biology, Temple University, Philadelphia, PA 19122-6078, USA; Department of Biology, Temple University, Philadelphia, PA 19122-6078, USA; Department of Biochemistry and Molecular Biology, The Pennsylvania State University, University Park, PA 16802, USA; Institute of Infectious Disease and Molecular Medicine, Department of Integrative Biomedical Sciences, University of Cape Town, Cape Town 7925, South Africa

**Keywords:** comparative genomics, codon models, purifying selection, evolutionary stasis, B-STILL

## Abstract

The quantification of genomic conservation has progressed from foundational statistical modeling of evolutionary rates to state-of-the-art deep learning architectures. However, a major resolution gap remains at the zero-rate origin, where standard selection inference tools fail to distinguish between sites that are invariant due to chance (stochastic invariance) or low substitution opportunity and those that are invariant due to extreme purifying selection. We present B-STILL (Bayesian Significance Test of Invariant Low Likelihoods), a hierarchical Bayesian framework designed to resolve the selective landscape of protein-coding genes near the zero-rate limit. By leveraging gene-level rate distributions (prior calibration) and modeling codon-site-specific substitution opportunities (determined by genetic-code degeneracy and nucleotide substitution biases), B-STILL quantifies the statistical significance of observed stasis. We define a rate-based stasis threshold to identify evolutionary stasis anchors (ESAs)—sites where the upper bound on the evolutionary rate is statistically constrained relative to the background rate of the gene due to extreme purifying selection. Validation against clinical and pathogen datasets confirms that ESAs are strong predictors of biological fitness and pathogenicity. Applying B-STILL across viral and mammalian genomes, we identify thousands of significantly clustered ESAs that map to known functional domains and uncharacterized structural motifs. These results establish B-STILL as a scalable, statistically rigorous framework for high-resolution genomic annotation, converting previously uninformative invariant sites into precise markers of extreme evolutionary constraint.

Significance Distinguishing genome positions under extreme purifying selection from those that remain unchanged by chance alone is a major challenge in evolutionary biology. We present B-STILL (Bayesian Significance Test of Invariant Low Likelihoods), a computational framework that differentiates these scenarios by modeling site-specific substitution opportunity. By analyzing viral and mammalian genomes, we demonstrate that B-STILL identifies evolutionary stasis anchors—highly conserved sites that serve as key indicators of critical functional and structural constraints.

## Introduction

Historically, invariable nucleotide sites have been viewed as uninformative for phylogenetic reconstruction, as positions lacking variation provide no signal for resolving tree topology. Consequently, these sites were often relegated to background covariates—most notably the “+I” parameter in general time reversible (GTR) + I + *Γ* models—designed to correct for rate heterogeneity and prevent the artificial underestimation of branch lengths. This approach, while computationally convenient, effectively acts as a black box that discards site-specific information, obscuring a primary signature of purifying selection: the absolute persistence of specific nucleotide states at a genomic position across deep time.

The field of genomic functional annotation has since transitioned through three distinct analytical epochs. The first was defined by the mathematical formalization of phylogenetic likelihoods, yielding foundational tools like phyloP (Davydov et al. 2010; Pollard et al. 2010) that provided essential, site-specific maps of purifying selection. While effective for identifying conserved regulatory elements, these methods often encounter a “significance plateau” at invariable sites in deep alignments. In the limit of absolute stasis—defined here as the complete absence of observed nucleotide substitutions at a site in the alignment—standard likelihood ratio tests (LRTs) saturate at a maximal significance ceiling. Under these conditions, LRTs lose the resolution necessary to distinguish between sites that are invariant due to low evolutionary opportunity (where substitutions are unlikely to occur under neutral evolution) and those that are invariant or nearly invariant due to extreme, site-specific purifying selection (evolutionary constraint). For a detailed technical review of existing methodologies and their respective limitations in the context of site-level conservation, see the Supplementary material. The second epoch expanded the dimensionality of conservation, utilizing sophisticated null models to identify multilayered and overlapping regulatory architectures (Sealfon et al. 2015). Currently, the field is entering a third epoch dominated by phylogeny-aware deep learning architectures and genomic language models (gLMs) such as GPN-Star (Ye et al. 2025; Avsec et al. 2026). These models shift the paradigm from raw nucleotide identity to “semantic matching,” capturing hidden regulatory grammar across hundreds of millions of years. However, these advanced neural networks often act as black boxes, making it difficult to isolate the underlying phylogenetic signal from complex biochemical and contextual features.

We present B-STILL (Bayesian Significance Test of Invariant Low Likelihoods), a framework that bridges these eras by resolving the selective landscape of protein-coding sequence data around the limit of absolute evolutionary stasis. Crucially, a site may appear invariant in an alignment for three distinct reasons: (i) it is under extreme purifying selection at the amino-acid level, where any nonsynonymous change is deleterious; (ii) it has low evolutionary opportunity (ie it is “evolutionarily cold”) due to local DNA repair dynamics, low tree length (insufficient evolutionary depth), or codon structure that restricts synonymous substitutions; or (iii) it is stochastically invariant, where substitutions could have occurred neutrally but did not by random chance. We define sites in the first category as evolutionary stasis anchors (ESAs). The core intuition of B-STILL is that the significance of an invariant site is conditioned on its cumulative substitution opportunity. By leveraging the site-specific and gene-specific synonymous substitution rate distributions as an internal control for the neutral background substitution rate, B-STILL distinguishes between sites that are invariant because they are evolutionarily cold and those that are truly anchored (absolute sequence stasis despite synonymous opportunity). By pooling information across the entire gene to estimate data-specific empirical priors, B-STILL calculates an empirical Bayes factor (EBF) that identifies ESAs as positions where strict sequence constraint is statistically anomalous relative to the global selective regime for the entire gene. To identify larger-scale signatures of constraint, we complement these site-level inferences with a nonparametric Hypergeometric Scan Statistic that detects regional *Stasis Clusters*—contiguous genomic intervals exhibiting a significantly high density of ESAs relative to the gene-wide background.

## Materials and Methods

### The B-STILL Framework

B-STILL is built upon the FUBAR framework (Murrell et al. 2013), which utilizes a Dirichlet process prior to model rate heterogeneity across sites. The underlying evolutionary process is modeled using a standard Muse–Gaut (MG94) codon model modified with a General Reversible (REV) nucleotide substitution matrix (Muse and Gaut 1994; Kosakovsky Pond and Frost 2005). The rate of substitution from codon *i* to codon *j* ($q_{ij}$) is defined by


(1) $$q_{ij}=\left\{ \begin{array}{cc} \alpha \theta_{n_{i},n_{j}}\pi_{n_{j}} & \text{synonymous substitution of one nucleotide},\\ \beta \theta_{n_{i},n_{j}}\pi_{n_{j}} & \text{nonsynonymous substitution of one nucleotide},\\ 0 & \text{multiple step substitution}, \end{array}\right.$$

where *α* and *β* represent the site-specific synonymous and nonsynonymous substitution rates, *θ* is the nucleotide exchangeability (relative to $\theta_{\mathrm{AG}}=\theta_{\mathrm{GA}}=1$, and *π* is the target nucleotide frequency. The equilibrium frequency over sense codons is obtained using the ${\text{CF}}_{3\times 4}$ estimator (Kosakovsky Pond et al. 2010). In the B-STILL implementation, the nucleotide exchangeability parameters (*θ*) and branch lengths are first estimated using a standard GTR model on the entire alignment to provide a robust background fit. These parameters are then held fixed during the subsequent Bayesian inference phase, allowing the model to focus exclusively on site-specific rate variation.

### Fixed-Grid Bayesian Inference (FUBAR)

To achieve computational efficiency, B-STILL follows the FUBAR approach of approximating the continuous rate distribution using a discrete K×K grid of (α,β) points. Let $\Theta =\{(\alpha_{k},\beta_{k}){\}}_{k=1}^{K^{2}}$ be the set of grid points. The likelihood of the data at site *s* given a specific grid point *k* is $L_{s}(\alpha_{k},\beta_{k})$, which is calculated using the standard Felsenstein’s pruning algorithm. FUBAR avoids the computational burden of traditional MCMC sampling over branch lengths and substitution parameters by leveraging this fixed grid. The prior probability of each grid point, $\pi =\{\pi_{k}{\}}_{k=1}^{K^{2}}$, is estimated by pooling information across all *N* sites in the alignment. In B-STILL, we typically employ a 0th order variational Bayes (VB0) approximation, which is mathematically equivalent to the expectation–maximization algorithm with a Dirichlet prior on the grid weights, or a collapsed Gibbs sampler to estimate the prior weights π that maximize the marginal log-likelihood:


(2) $$L(\pi )=\sum_{s=1}^{N}\ln\;\left(\sum_{k=1}^{K^{2}}\pi_{k}L_{s}(\alpha_{k},\beta_{k})\right)$$

Once the gene-wide prior π is established, the posterior probability of site *s* belonging to rate regime *k* is calculated as


(3) $$P(k\;|\;{\text{Data}}_{s})=\frac{\pi_{k}L_{s}(\alpha_{k},\beta_{k})}{\sum_{\;j=1}^{K^{2}}\pi_{j}L_{s}(\alpha_{j},\beta_{j})}$$

This hierarchical sharing allows B-STILL to calibrate the surprise of invariance at a single site against the background frequency of conservation observed across the entire gene.

Standard selection scans can have limited resolution at the boundary of zero substitutions because linear or log-spaced grids lack the density required to resolve the likelihood surface near the origin. B-STILL addresses this by implementing a high-resolution quadratic grid in the near-zero regime. For a grid of *K* points per dimension, the rates for the *k*th point are defined as


(4) $$r_{k}={\left(\frac{k}{K_{\mathrm{neg}}-1}\right)}^{2}\times R_{\max}$$

This quadratic clustering provides the statistical sensitivity needed to distinguish between near-zero purifying selection and absolute evolutionary immobilization.

The primary statistical metric in B-STILL is the EBF, which quantifies the surprise of observing a specific selective regime relative to the gene-wide prior:

1. **Exact invariance (α=0,β=0)**: Quantifies evidence for absolute sequence constraint.
2. **Synonymous stasis (${\mathrm{EBF}}_{\mathrm{syn}}$, α=0)**: Quantifies evidence for constraint at the nucleotide level, regardless of the nonsynonymous rate.
3. **Nonsynonymous invariance (β=0)**: Quantifies evidence for constraint at the protein level, regardless of the synonymous rate.
4. **Proximal Stasis (${\mathrm{EBF}}_{\mathrm{prox}}$, E[S]<X)**: This metric is defined as the posterior probability mass concentrated on grid points (α,β) where the total expected number of substitutions along the gene tree is less than or equal to a fixed threshold (*X*, tunable by the user). The default value of X=0.5 represents a conservative, biologically intuitive threshold. Under a Poisson process, if the expected number of substitutions is E[S]≤0.5, the probability of observing zero substitutions is $e^{-0.5}\approx 60.6\%$, meaning that a neutral site is expected to remain invariant or nearly invariant over the evolutionary history of the gene. By defining stasis relative to expected substitutions (E[S]) rather than raw rate parameters, the boundary rescales in rate space based on tree length (*T*): $(\alpha \cdot d_{s}+\beta \cdot d_{ns})\leq X/T$. This ensures that B-STILL automatically controls false positives in shallow alignments (compressing EBFs toward 1.0) while maintaining high sensitivity in deep alignments. By explicitly defining a radius of stasis around the origin, B-STILL distinguishes between stochastic invariance and bona fide functional constraint.

Unless explicitly defined otherwise, the results and discussion presented herein focus exclusively on Proximal Stasis (${\mathrm{EBF}}_{\mathrm{prox}}$). The EBF for a state/event *S* is calculated as


(5) $$\mathrm{EBF}(S)=\frac{P(S\;|\;D)/(1-P(S\;|\;D))}{P(S)/(1-P(S))}$$

representing the ratio of posterior to prior odds.

### Hypergeometric Scan Statistic for Identifying Stasis Clusters

To identify unusually dense clusters of ESAs within individual coding regions, we implemented a nonparametric clustering algorithm based on an exact hypergeometric scan statistic. Under the null hypothesis ($H_{0}$), we assume that ESAs are distributed randomly across the sequence without spatial preference. The gene is treated as a finite population of *L* possible codon positions containing exactly *K* high-confidence ESA sites (eg EBF≥10).

We define a potential *Stasis Cluster* as any genomic interval $\left[x_{i},x_{j}\right]$ anchored by groups of high-confidence ESAs. For every interval, let *k* be the number of observed ESAs and $d=x_{j}-x_{i}+1$ be the span in codons. The local *p*-value is calculated using the upper tail of the hypergeometric distribution:


(6) $$p_{\mathrm{local}}(k,d)=\sum_{m=k}^{d}\frac{\left(\begin{array}{c} K\\ m \end{array}\right)\left(\begin{array}{c} L-K\\ d-m \end{array}\right)}{\left(\begin{array}{c} L\\ d \end{array}\right)}$$

This model accounts for sampling without replacement from a finite population of genomic sites, which is essential when the density of ESAs (K/L) is nonnegligible. To control the family-wise error rate (FWER), we performed a permutation test. For each gene, we generated 10,000 permuted datasets by shuffling the positions of the *K* ESAs. The 5th percentile of the null distribution of minimum local *p*-values was defined as the gene-specific critical significance threshold. Overlapping significant segments were merged into single continuous intervals to demarcate the regional bounds of individual Stasis Clusters.

### Regional Modeling: Comparison with the PHAST Framework

The B-STILL regional scan methodology represents a conceptual departure from established conservation-mapping frameworks such as PHAST (eg phastCons). While phastCons utilizes a hidden Markov model to identify conserved elements by modeling transitions between “conserved” and “nonconserved” states, it relies on regional rate smoothing to define block boundaries. In contrast, B-STILL identifies Stasis Clusters using a discrete point-process model. By anchoring clusters to specific ESAs, B-STILL provides spatial resolution for identifying multilayered features—such as overlapping ORFs or RNA structures—where the requirement for sequence immutability is concentrated in narrow, high-density intervals.

### HIV-1 Structural Mapping and Analysis

To evaluate the protein structural context of ESAs, we projected site-level EBF results onto the crystal structure of the HIV-1 RT heterodimer (PDB: 1RTD; Huang et al. 1998). Residue-to-residue mapping between the alignment consensus and the PDB sequence was established using global protein alignment (BLOSUM62). Invariant residues were visualized as spheres using the PyMOL Molecular Graphics System (Schrödinger and DeLano 2020). We specifically analyzed the spatial clustering of ESAs within the catalytic palm subdomain, encompassing the Asp110–Asp185–Asp186 triad, and the primer-grip motif (residues 227–235).

### Simulation Benchmarks and Power Analysis

To evaluate the statistical properties of the B-STILL framework, we performed power simulations using a mosaic sequence design across 90 distinct evolutionary scenarios, totaling 1,800 replicates. Each simulated alignment was constructed by splicing two selective regimes: a Neutral partition comprising between 90% and 99% of the sites, and a Stasis partition comprising the remaining 1–10%. Data were generated using the simulate tool in HyPhy, with the neutral partition evolving under gene-specific synonymous and nonsynonymous rates (α,β) estimated from six empirical datasets: HIV-1 RT (T=6.42, *T* = tree length in expected substitutions per site), plant RuBisCO (T=10.99, rbcL), SARS-CoV-2 Spike (T=0.12), mammalian beta-globin (T=2.25, bglobin), Camelid VHH (T=14.13), and encephalitis virus envelope (T=0.83). For each empirical background, we evaluated performance across five tree-scaling factors (0.1×, 0.5×, 1.0×, 2.0×, and 5.0×) and three stasis-injection proportions (0.01,0.05,and0.10), generating 10 replicates per scenario. The stasis partition was simulated by scaling the background tree to model an approximate stasis selective regime, where the upper bound on permitted evolutionary change is lower than the gene-wide average (E[S]≤0.5). We also included a strict null scenario where 100% of the sites evolved under the background empirical rates without stasis injection to measure the intrinsic FPR. Replicates were analyzed using B-STILL with a Proximal Stasis threshold of EBF≥10, and performance was assessed by comparing the inferred ESAs to the coordinates of the spliced stasis partition. This design tests the framework’s ability to distinguish functional sequence constraint from stochastic invariance across a spectrum of evolutionary depths and ESA densities.

### Gene Selection for Mammalian Exome Analysis

To evaluate the performance of B-STILL on human-relevant pathology, we targeted a definitive set of genes harboring well-documented, disease-causing synonymous variants. Candidate genes were selected based on a comprehensive review of the clinical genetics literature and databases (eg ClinVar, HGMD), focusing on positions where synonymous single-nucleotide variants have been definitively established as pathogenic drivers through in vitro or in vivo validation.

The selection process prioritized four primary molecular mechanisms of synonymous pathogenicity: (1) disruption of pre-mRNA splicing networks (eg MSH2, TP53, BRCA1), (2) perturbation of cotranslational protein folding and translation kinetics (eg CFTR, F9), (3) alteration of mRNA thermodynamic stability (eg DRD2), and (4) destruction or creation of microRNA binding sites (eg IRGM). This yielded a validation panel of 38 selected genes representing diverse functional classes, including tumor suppressors, coagulation factors, and metabolic enzymes.

### Comparative Benchmarking Against PhyloP

To compare the B-STILL codon-aware framework with phyloP, we conducted a head-to-head comparison against phyloP (Pollard et al. 2010) using a validation panel of 38 representative mammalian genes. This panel was selected to encompass a spectrum of selective regimes, ranging from ultra-conserved housekeeping genes (eg CALM1) to fast-evolving antiviral and surface proteins (eg APOBEC3D, MUC1), as well as established clinical targets (TP53, BRCA1, CFTR). To ensure a comparison, phyloP was executed on the identical 120-species mammalian alignments (Hecker and Hiller 2020) and neutral phylogenetic backgrounds used for the B-STILL analysis. Specifically, for each gene, we first estimated a synonymous-aware neutral tree using the FitMG94 framework (FitMG94.bf) (Kosakovsky Pond and Frost 2005), extracting the expected synonymous substitutions per nucleotide site across the entire phylogeny. This tree was utilized as a fixed constraint in phyloFit (Siepel et al. 2005) to estimate gene-specific GTR substitution parameters and equilibrium frequencies (--estimate-freqs) while holding branch lengths constant (--no-opt branches). Site-specific conservation and acceleration scores were subsequently calculated using phyloP with the LRT (--method LRT) in CONACC mode.

### Dark Proteome Analysis

To evaluate the utility of B-STILL for the functional annotation of uncharacterized proteins, we performed a proteome-wide scan of the “dark proteome”—defined here as the subset of the human genome consisting of uncharacterized ORFs and poorly characterized gene families. Our analysis targeted 815 genes from the 120-species mammalian alignment (Hecker and Hiller 2020), including the C*orf (284 genes), FAM (228 genes), TMEM (272 genes), and KIAA (31 genes) nomenclature groups. These genes were prioritized based on the absence of experimentally validated biochemical functions or detailed structural characterization in major databases (eg UniProt, PDB). For each gene, we identified ESAs and spatial clusters (EBF≥100, increased stringency). To investigate the structural implications of these ESAs, site-specific Bayesian factors were mapped onto AlphaFold-predicted 3D structures (Jumper et al. 2021). The spatial distribution of selective constraint was visualized by substituting the B-factor column of the corresponding PDB files with $\log_{10}\;({\mathrm{EBF}}_{\mathrm{prox}})$. This approach allowed for the identification of physically clustered ESAs in three-dimensional space, providing a data-driven strategy for prioritizing structural hubs and potential interaction interfaces in proteins that lack experimental characterization.

### Validation Against Human Population Variation

We validated the biological relevance of mammalian ESAs by cross-referencing our phylogenetic inferences with human population-level variation from the gnomAD database (v4.1.1; Chen et al. 2024). For each analyzed mammalian gene (a subset of N=68), we mapped human protein coordinates to the 120-species alignment consensus (Hecker and Hiller 2020) using global protein alignment (BLOSUM62) and the HGVSp nomenclature (Hart et al. 2024). We retrieved nonsynonymous and synonymous variants and quantified the relationship between B-STILL EBFs and human allele frequencies using the Spearman rank correlation coefficient. To assess clinical utility, we performed a receiver operating characteristic analysis using pathogenic and benign variants from the ClinVar database (Landrum et al. 2018) for a further subset of these genes chosen for known clinical significance (N=18). For this analysis, ClinVar clinical significance was used as the binary classification target (pathogenic and likely pathogenic variants were assigned a value of 1; Benign and likely Benign variants were assigned a value of 0). We utilized B-STILL EBFs as continuous predictors to assess their ability to distinguish between clinically confirmed deleterious substitutions and neutral variation. Specifically, we calculated the AUROC using ${\text{EBF}}_{\mathrm{prox}}$ for nonsynonymous variants and ${\text{EBF}}_{\mathrm{syn}}$ for synonymous variants. Only high-confidence variants (reviewed by an expert panel or with multiple submitters and no conflicting interpretations) were included in the validation panel to ensure a rigorous clinical ground truth.

### REVEL Correlation and Clinical Benchmarking

To further evaluate the relationship between B-STILL EBFs and established clinical pathogenicity predictors, we performed a genome-scale comparison with the REVEL (Rare Exome Variant Ensemble Learner) ensemble method (Ioannidis et al. 2016). We selected a validation panel of 85 genes by identifying the top 100 fastest-evolving genes in our mammalian dataset (ranked by total tree length) and filtering for those with available nonsynonymous variant scores in the MyVariant.info database (Louter et al. 2016). This strategy targeted high-divergence gene families—including zinc fingers (ZNF), olfactory receptors (OR), and taste receptors (TAS2R)—to evaluate B-STILL’s performance in selective regimes with high substitution opportunity. The resulting dataset encompassed 34,206 individual nonsynonymous variant positions. REVEL scores and protein-level coordinates were retrieved programmatically using the MyVariant.info REST API. To ensure precise coordinate integration, human protein positions were mapped to the 120-mammal MSA using translated hg38 sequences, accounting for local gaps and indels.

### Analysis of Overlapping Reading Frames

To evaluate the performance of B-STILL in detecting multilayered functional constraints, we analyzed 111 viral protein-coding alignments from the FRESCO dataset (Sealfon et al. 2015). For each alignment, we computed site-specific EBFs for proximal stasis (α≈0,β≈0). To validate these signals, we curated reference genomes from the NCBI Virus RefSeq database (eg NC_004102 for hepatitis C, NC_011505 for rotavirus A). Annotated overlapping reading frames were defined as any two CDS features sharing genomic coordinates but translated in different frames or on opposite strands. Nucleotide-aware projection (BLOSUM62) was used to map RefSeq coordinates to alignment indices, accounting for gaps and frame-shifts.

### Implementation and Availability

B-STILL is implemented as a standard analysis module within the HyPhy software package (version 2.5.95 or later) using the HyPhy Batch Language (HBL). The implementation leverages HyPhy’s high-performance likelihood engine and supports both traditional grid-based posterior estimation (via a Collapsed Gibbs sampler) and rapid 0th order variational Bayes inference (VB0). The framework introduces a configurable radius-threshold parameter, which corresponds to the *X* value described in the methodology. This parameter allows users to define proximal constraint in terms of total evolutionary surprise across the tree. For example, setting X=0.5 effectively classifies any rate regime expected to produce fewer than one substitution across the entire phylogeny as belonging to the proximal invariant state. This approach is critical for distinguishing between sites that are “historically invariant” (zero substitutions observed) and those that are “effectively invariant” (substitution rates significantly below the gene-induced expectation even if rare substitutions are present). Resulting EBFs and model parameters are exported in a structured JSON format, compatible with the HyPhy vision ecosystem for interactive visualization. Significant regional Stasis Cluster signals were identified using a standalone Python tool, infer_stasis_clusters.py. This tool implements the exact Hypergeometric Scan Statistic described above, performing 10,000 permutations per gene to determine gene-specific critical *p*-value thresholds for FWER control. The source code for B-STILL and the Hypergeometric Scan Statistic is available under the MIT license at https://github.com/veg/hyphy and https://github.com/veg/b-still. B-STILL is also available through a user-friendly web interface on Datamonkey v3 (v3.datamonkey.org) and as a tool within the Galaxy Project ecosystem.

## Results

### Methods Overview

**Fig. 1.**
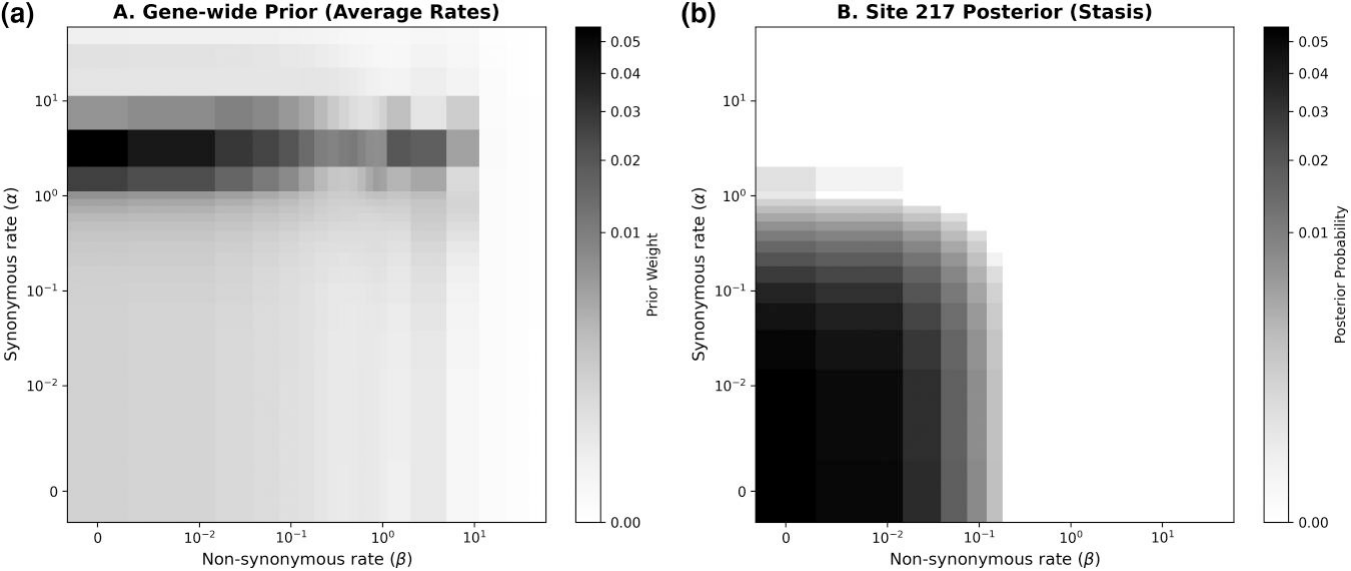
Bayesian resolution of evolutionary stasis in HIV-1 reverse transcriptase. a) The gene-wide prior distribution of synonymous (*α*) and nonsynonymous (*β*) substitution rates, representing the average selective landscape across the entire gene. b) The posterior distribution for site 217, showing the concentration of probability mass at the (0,0) origin. This transition from a broad prior to a peaked posterior at the origin is the hallmark signature of an ESA.

To facilitate the interpretation of our empirical findings, we outline the mathematical and conceptual architecture of the B-STILL framework (see Fig. 1 for a conceptual overview). B-STILL is developed as an empirical Bayes extension of codon-based phylogenetic models. Rather than evaluating each codon site in isolation, B-STILL pools information across the entire gene alignment to estimate a joint empirical prior distribution of synonymous (*α*) and nonsynonymous (*β*) substitution rates. This prior is discretized over a grid of rate values, approximating the continuous distribution of selective pressures across the gene. B-STILL builds directly upon the fast, unconstrained Bayesian approximation (FUBAR) framework (Murrell et al. 2013), leveraging its discrete grid-based likelihood and variational Bayes estimators. While FUBAR and standard Muse–Gaut (MG94) codon models account for GTR nucleotide substitution biases and transition/transversion rates as standard terrain, a distinguishing feature of B-STILL is the parameterization and evaluation of the near-zero-rate origin to isolate extreme purifying selection from stochastic rate variation.

For each site, B-STILL combines the alignment data with the gene-wide prior to compute the joint posterior probability distribution of the rate parameters (α,β). We define a site as being in a state of stasis (an ESA) if the total expected number of substitutions along the tree is less than or equal to a user-specified threshold, E[S]≤X, with a default of X=0.5. Formally, the expected number of substitutions is computed as $E[S]=(\alpha \cdot d_{s}+\beta \cdot d_{\mathrm{ns}})T$, where *T* represents the tree length (the sum of branch lengths in expected substitutions per site), and $d_{s}$ and $d_{\mathrm{ns}}$ denote the site-specific synonymous and nonsynonymous weighting factors (functions of other model parameters). To quantify the statistical evidence for stasis, B-STILL computes an EBF, which is the ratio of the posterior odds to the prior odds of a site falling within the stasis regime (E[S]≤X). By conditioning the stasis boundary on expected substitutions, B-STILL naturally accounts for the total evolutionary depth of the alignment: in shallow trees where the prior expectation of substitutions is low, stasis is not surprising and EBFs remain low; in deep trees where neutral substitutions are expected, the absence of change yields high EBFs. B-STILL partitions total constraint into synonymous stasis (${\text{EBF}}_{\mathrm{syn}}$), where nonsynonymous rates are unconstrained but synonymous rates are near-zero, and proximal stasis (${\text{EBF}}_{\mathrm{prox}}$), where both rates are near-zero.

### HIV-1 Reverse Transcriptase Case Study

The B-STILL framework resolves site-specific selective regimes by leveraging both gene-wide information and local substitution opportunity. This is illustrated by comparing the global rate distribution (the prior) with the inferred posterior distribution for a site under extreme evolutionary stasis (Fig. 1). While the gene-wide prior for HIV-1 reverse transcriptase (RT) reflects a range of synonymous and nonsynonymous rates (Fig. 1a), B-STILL identifies sites where the local data effectively pulls the probability mass toward the (0,0) origin (Fig. 1b). Our results reveal that not all invariant sites are equal. The EBF effectively measures the surprise of observed stasis relative to a codon-aware gene-wide baseline. A codon with high synonymous redundancy, such as Proline (CC*), faces a significantly higher expected rate of synonymous drift than a codon with low redundancy, such as tyrosine (TA[T/C]). Under a neutral model, the probability of observing zero substitutions at a site with high synonymous opportunity is relatively smaller compared to a site where substitution targets are restricted by the genetic code. Consequently, B-STILL yields a substantially higher EBF for invariance at highly redundant codons, as their stasis represents a more substantial deviation from the gene-wide expectation.

Furthermore, the method accounts for the underlying nucleotide substitution matrix. Under the GTR model, if the inferred exchangeability parameters indicate a strong transition/transversion bias (or other specific substitution preferences), a codon whose synonymous targets require a more frequent substitution type (such as a transition) is expected to vary more than one requiring a less frequent type (such as a transversion). For example, comparing site 217 and site 232 in RT illustrates this resolution. Site 217 (proline, CCA) resides in a 4-fold degenerate block with multiple high-probability transition paths to synonymy. Despite the presence of mixed bases (CCW and CCM), which represent synonymous ambiguities, the total lack of nonsynonymous change is statistically significant (${\text{EBF}}_{217}=1,986.41$). In contrast, site 232 (tyrosine, TAT) has a much narrower synonymous neighborhood (S=1). While it also exhibits an ambiguity (TAY), its stasis is less surprising under the hierarchical prior, resulting in a lower, albeit still significant, EBF (107.39).

By explicitly calculating the synonymous and nonsynonymous rate parameters, we can partition the total constraint into its respective components, revealing sites that are under intense nonsynonymous purifying selection despite being synonymous neutral. Projection of invariant sites onto the RT crystal structure further corroborates their functional relevance. Invariant residues cluster in catalytically and structurally critical regions, including the palm subdomain and the primer-grip motif (Fig. S1). This spatial enrichment provides independent structural validation that B-STILL proximal-constraint calls are associated with functional constraint.

As an example of extrinsic validation, we reasoned that sites inferred as highly constrained by B-STILL from this training dataset would exhibit lower variability in a large population sample. Indeed, examining a population database of over 175,000 HIV-1 sequences (Stanford HIVDB; Rhee et al. 2003), we found that evolutionary stasis is a predictor of human population-level variability: codons with higher B-STILL EBFs had lower frequencies of amino-acid substitutions ($\rho =-0.3271,p<10^{-9}$). Significant proximal ESAs identified in the RT alignment (ie those with associated EBFs >10) are summarized in Table S1.

### Simulation Benchmarks: Specificity and Depth-Dependent Power

**Fig. 2.**
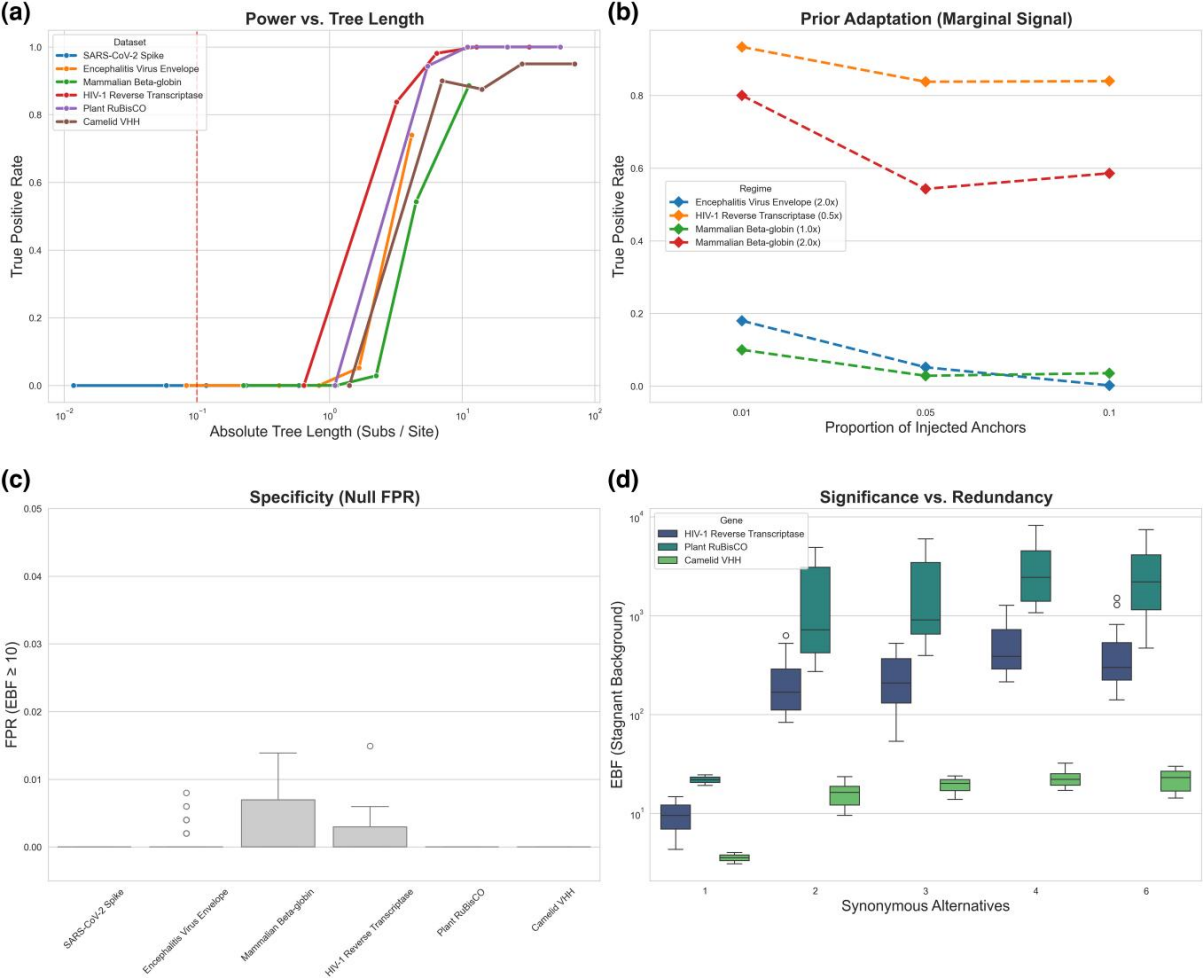
B-STILL performance summary across 1,800 simulated datasets. a) Detection power (TPR) as a function of absolute tree length (at 5% injected ESA density). Sensitivity scales log-linearly with total substitution count, reaching near saturation in deep phylogenies (T>5). b) Power vs. anchor density. Power decreases as the proportion of ESAs increases in marginal signal regimes (eg encephalitis virus envelope and mammalian beta-globin), reflecting the adaptation of the Bayesian prior to a more evolutionarily stagnant background. c) FPR control remains robustly below 1% across all datasets. d) Significance vs. codon redundancy. Absolute stasis is statistically more significant at redundant codons.

Benchmarks across 1,800 nucleotide sequence datasets that were simulated using the phylogenies of six representative genes under three different scenarios, confirm that B-STILL is conservative, maintaining a null false-positive rate (FPR) ≪1% across all datasets (Fig. 2c and Table S2). Additionally, as shown in Fig. 2c, the FPR of B-STILL remains robustly below 1% (typically close to 0.05%) across all simulated datasets, demonstrating strict type I error control regardless of the dataset depth or model parameters.

In the strict null simulation scenario (100% background sites), the framework identified near-zero false positives, confirming that the Bayesian prior adapts to the background substitution process. In datasets simulated using the very shallow SARS-CoV-2 Spike phylogeny (T=0.12), B-STILL correctly identified zero ESAs (proximal EBF for X=0.5 expected substitutions per site), confirming that the absence of variation in young lineages is correctly deweighted as statistically uninformative (Fig. 2a). These benchmarks reveal that B-STILL utilizes the redundancy of the genetic code as an internal control for substitution opportunity. We observed that absolute stasis is statistically more significant at redundant codons (eg 4-fold proline) than at low-redundancy ones (eg methionine), reflecting the greater deviation from expectation when substitutions are absent despite opportunities for synonymous variation (Fig. 2d).

The power to detect ESAs is driven by evolutionary contrast. We evaluated performance across datasets that were simulated using six empirical phylogenies representing a range of different tree lengths (*T*): HIV-1 RT (T=6.42), RuBisCO (T=10.99), SARS-CoV-2 Spike (T=0.12), mammalian beta-globin (T=2.25), Camelid VHH (T=14.13), and encephalitis virus envelope (T=0.83). As illustrated in Fig. 2a, the detection power (TPR) is highly dependent on tree length and only becomes high (sensitivity approaching 100%) when the tree length becomes quite long (T≥5.0, such as in HIV-1 RT, RuBisCO, and Camelid VHH). For shallow or moderate tree lengths (T<2.0), power is severely restricted due to the lack of sufficient evolutionary depth to generate contrast against stochastic invariance. For deep phylogenies (HIV-1 RT, RuBisCO, Camelid VHH), B-STILL identifies ESAs with high sensitivity (TPR ≈100%) (Fig. 2a) while maintaining high specificity (Fig. 2c). For phylogenies of moderate depth, such as beta-globin and encephalitis envelope, TPR scales log-linearly with tree length, reaching >70% when background variation is scaled to 5× the empirical tree depth.

Sensitivity benchmarks reveal that self-calibration through prior adaptation is most pronounced in the marginal signal regime (Fig. 2b). In datasets simulated using phylogenies of moderate depth (encephalitis virus envelope and mammalian beta-globin), we observe a consistent decrease in detection power as the proportion of ESAs increases. Notably, Fig. 2b also reveals a substantial gap in power between different empirical backgrounds of moderate depth (eg mammalian beta-globin at T=2.25 vs. encephalitis virus envelope at T=0.83). This gap highlights how critical small differences in tree length are for detection sensitivity when the total evolutionary depth is in the marginal regime, with the deeper beta-globin tree consistently yielding double the power of the envelope tree across all anchor densities. This phenomenon demonstrates the hierarchical prior’s role in governing conservatism: when stasis is rare, any invariant site is highly statistically surprising, but as the gene-wide background becomes more stagnant, stasis at any given site becomes less surprising and the threshold for significance naturally increases. This effect disappears in datasets simulated using deep phylogenies (HIV-1 RT and RuBisCO), where the signal of functional immobilization is strong enough to saturate detection power regardless of the density of ESAs.

Finally, as suggested by the HIV-1 RT analysis, synonymous codon redundancy partially informs the detection of ESAs. For sites simulated under stasis, levels of statistical surprise compared to the background of the gene were higher for codons with higher synonymous redundancy (Fig. 2d). This finding confirms the intuition that when a highly synonymously redundant site harbors an unexpectedly small number of substitutions, it provides stronger statistical evidence of constraint than when the same behavior is seen at a less redundant site.

### Global Scan of the Mammalian Exome

**Fig. 3.**
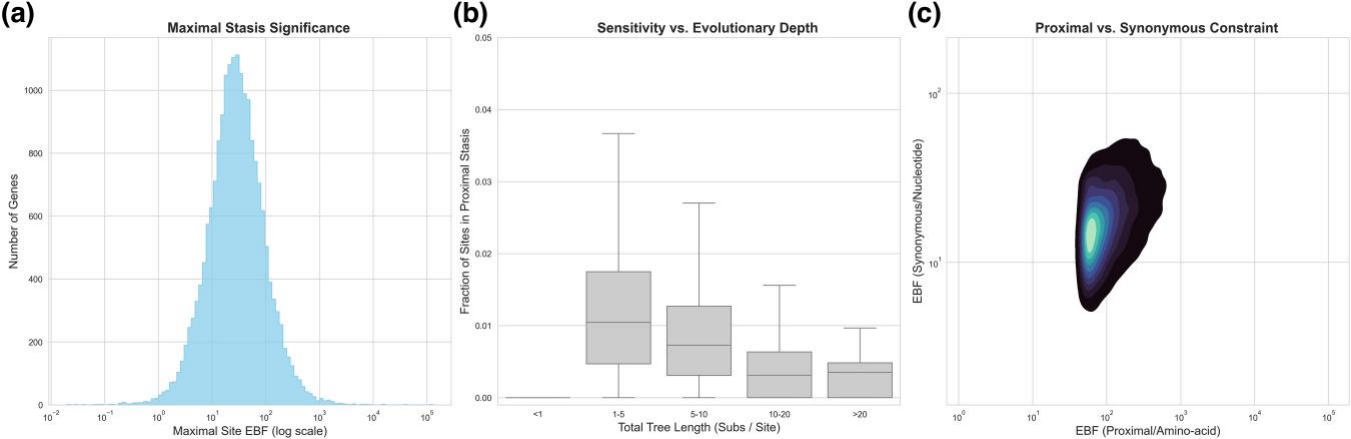
Patterns of evolutionary stasis across 19,117 mammalian genes. a) Distribution of maximal stasis significance. Isolated ESAs in variable genes reach EBF values ($>10^{6}$). b) Detection sensitivity vs. depth. Signals of evolutionary stasis are negligible in shallow phylogenies (T<1) and peak in phylogenies of moderate depth (T=1–5). c) Selective hierarchy. Absolute sequence constraint (α,β≈0) provides a more powerful signal of deviation from expectation than synonymous-only constraint.

We applied B-STILL to 19,117 protein-coding genes from the 120-species mammalian alignment (Hecker and Hiller 2020), totaling 10,990,498 codon sites (Hecker and Hiller 2020). This large-scale scan identifies sites where stasis is anomalous relative to the gene-wide selective background, providing a high-resolution map of ESAs across the proteome (Fig. 3). As shown in Fig. 3a, the distribution of maximal stasis significance shows that the majority of mammalian genes harbor at least one highly significant ESA. In variable genes under active diversifying pressure, isolated ESAs can reach EBF values exceeding $10^{6}$, highlighting positions under extreme purifying selection despite a globally variable background.

The prevalence of ESAs exhibits a characteristic nonmonotonic relationship with evolutionary depth. Our results demonstrate that detection sensitivity is negligible in shallow phylogenies (tree length T<1 substitution per site, Fig. 3b), where evolutionary stasis is statistically expected. The detectability of ESAs peaks in phylogenies of moderate depth (*T* between 1 and 5 substitutions per site, median proportion of static sites = 1.04%), providing the optimal contrast for resolving selective constraint across the proteome. Notably, the proportion of identified ESAs declines progressively as evolutionary depth increases, falling to 0.35% in the deepest phylogenies (T>20). However, the intensity of stasis significance (maximal site EBF) scales positively with tree length, with the mean maximal EBF rising from 2.44 in shallow phylogenies to over 4,500 in the deepest phylogenies (not shown). This trend confirms that while the requirement for absolute sequence immutability through deep time is rare, the observation of such stasis in high-divergence alignments provides a powerful signal of functional evolutionary constraint.

Across the mammalian exome, 151,146 sites (1.38%) exhibit significant evidence of proximal stasis (${\text{EBF}}_{\mathrm{prox}}\geq 10$), while only 74,080 sites (0.67%) achieve the same threshold for synonymous-only constraint (${\text{EBF}}_{\mathrm{syn}}\geq 10$). This characteristic hierarchy in significance reflects the framework’s focus on statistical surprise. Because the prior expectation for absolute sequence constraint is an order of magnitude lower than for synonymous stasis, the observation of a completely invariant site provides a significantly larger signal of deviation from expectation (Fig. 3c).

The prevalence of detectable ESAs is nonuniformly distributed across the genome. While the average gene contains approximately 35 significant proximal ESAs, a subset of genes—often those involved in core developmental processes, RNA regulation, or structural stabilization—contains “islands” of extreme conservation where every site is highly constrained. As predicted by the B-STILL hierarchical prior, the statistical “surprise” of a given invariant site is strongly dependent on the background substitution rate of the gene. In fast-evolving genes such as olfactory receptors or mucins, isolated invariant sites achieve high EBF values ($>10^{6}$), representing ESAs associated with critical structural or functional roles despite substantial diversifying pressure on the rest of the protein (eg OR4N5 Site 169, $\mathrm{EBF}=8.03\times 10^{8}$). Conversely, in ultra-conserved genes like histones or calmodulin, the high prior expectation of invariance deweights individual sites, appropriately identifying them as part of a globally constrained architecture rather than specific anomalous ESAs. These findings demonstrate that B-STILL distinguishes between uninformative sequence conservation and the operational core of protein function.

### Validation Against Human Variation and Clinical Ground Truth

We cross-referenced the sites found by B-STILL in the scan of a panel of 68 mammalian genes (see Materials and Methods) against both degrees of variation at those sites in the human population (using gnomAD v4.1.1; Chen et al. 2024), and evidence of variants at the sites having pathological consequences in humans using the ClinVar database (Landrum et al. 2018) and the REVEL clinical pathogenicity predictor (Ioannidis et al. 2016). Our analysis revealed statistically significant negative correlations between mammalian EBFs and human allele frequencies for the majority of genes, with the strongest predictive power observed for genes like VHL and F9 (Table 1).

**Table 1.** Validation of B-STILL proximal EBFs against human population variation (gnomAD v4.1)

Gene	Sites	Spearman *ρ*	*p*-value
VHL	214	− 0.357	7.69e−08
G6PD	516	− 0.276	1.92e−10
F9	462	− 0.242	1.35e−07
CDH1	883	− 0.218	6.29e−11
IL2RG	370	− 0.215	3.09e−05
TP53	394	− 0.205	4.27e−05
F8	2,352	− 0.204	1.29e−23
MSH6	1,361	− 0.200	1.05e−13
CHRNA1	458	− 0.197	2.29e−05
BTK	660	− 0.189	1.00e−06
AASS	927	− 0.188	8.21e−09
DEPDC5	1,604	− 0.186	6.72e−14
GRIN2A	1,465	− 0.176	1.12e−11
LMNA	665	− 0.175	5.69e−06
HEXA	530	− 0.168	1.00e−04
IRGM	182	− 0.165	0.026
RB1	928	− 0.157	1.63e−06
ATM	3,057	− 0.152	2.68e−17
HPRT1	219	− 0.129	0.056
GLA	430	− 0.123	0.011

*ρ* represents the Spearman correlation between site-level EBF and the sum of allele frequencies at that site. Top 20 genes by correlation magnitude are shown.

B-STILL EBFs demonstrate strong predictive power with respect to predicting clinical pathogenicity (Fig. S2) in a subset of 18 genes selected for harboring known pathogenic variants. For nonsynonymous variants, the B-STILL framework yields an aggregate area under the curve (AUROC) of 0.65 (mean per gene AUROC=0.71). More notably, B-STILL identifies evidence of clinically relevant synonymous variants, achieving an aggregate AUROC of 0.88 (mean per gene AUROC=0.89) using ${\text{EBF}}_{\mathrm{syn}}$, identifying evolutionary constraints on substitutions in regulatory sequences that are embedded within coding regions: constraints that are not typically captured by models that focus exclusively on protein sequences. We acknowledge that ClinVar incorporates sequence conservation as a criterion in its pathogenicity classification guidelines (Richards et al. 2015). Consequently, our validation against ClinVar is not entirely independent, as the B-STILL quantification of evolutionary stasis shares a common signal with the database’s annotations. Nevertheless, the high predictive performance (particularly for synonymous variants where standard nucleotide-level and protein-level conservation scores are uninformative) confirms B-STILL’s ability to extract and refine this selective signal directly from sequence alignments.

**Fig. 4.**
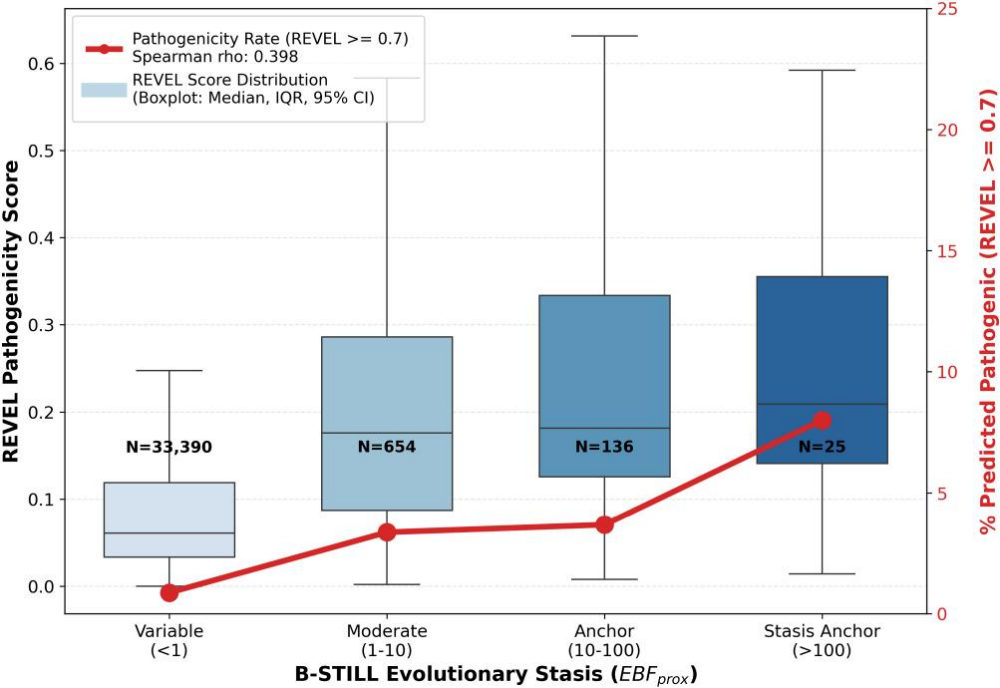
Genome-scale concordance between B-STILL inferred evolutionary stasis and REVEL pathogenicity scores (N=34,206 positions). The *X*-axis groups variants into four discrete stasis categories based on site-level ${\mathrm{EBF}}_{\mathrm{prox}}$. The left *Y*-axis (boxplots) shows the distribution of continuous REVEL scores for each category, with the sample size (*N*) indicated at the bottom. The right *Y*-axis (line) tracks the pathogenicity rate, defined as the percentage of variants within each category exceeding the high-confidence REVEL threshold (≥0.70). *ρ* represents the Spearman rank correlation coefficient, demonstrating an alignment between deep-time sequence constraint and modern clinical consensus.

For the panel of N=85 reference genes, B-STILL EBFs show a strong positive correlation with REVEL pathogenicity scores ($\rho =0.40,p<10^{-300}$, Fig. 4). This concordance is noteworthy given that ESAs represent only the most extreme subset of the proteome: a conservative marker that captures only a fraction of the total landscape of clinical pathology. Furthermore, while REVEL is explicitly designed to score nonsynonymous substitutions and cannot therefore evaluate nonprotein-altering variation, B-STILL leverages a single phylogenetic signal of absolute sequence constraint that applies to both nonsynonymous and synonymous positions. Despite its limited scope relative to REVEL’s 13-feature ensemble, B-STILL correctly identifies a shared functional core of genome sites where extreme evolutionary stasis and clinical consensus align, while also highlighting “Hidden Anchors” where intense purifying selection precedes or complements clinical consensus. For instance, at site 351 of FGFR3 where mutations are known to cause hypochondroplasia (Yao et al. 2019), B-STILL identifies a high-confidence ESA (EBF=215.9) while the REVEL score remains low (0.15). Additionally, B-STILL identifies critical clinical constraint at sites where substitutions are strictly synonymous in humans, such as TP53 site 125 (${\text{EBF}}_{\mathrm{prox}}=110$) and CFTR site 1,239 (${\text{EBF}}_{\mathrm{syn}}=109$), which map to documented regulatory pathogenic variants (Molinski et al. 2014; Pinto et al. 2022).

### Identifying Regional Footprints of Overlapping Reading Frames in Viral Genomes

**Fig. 5.**
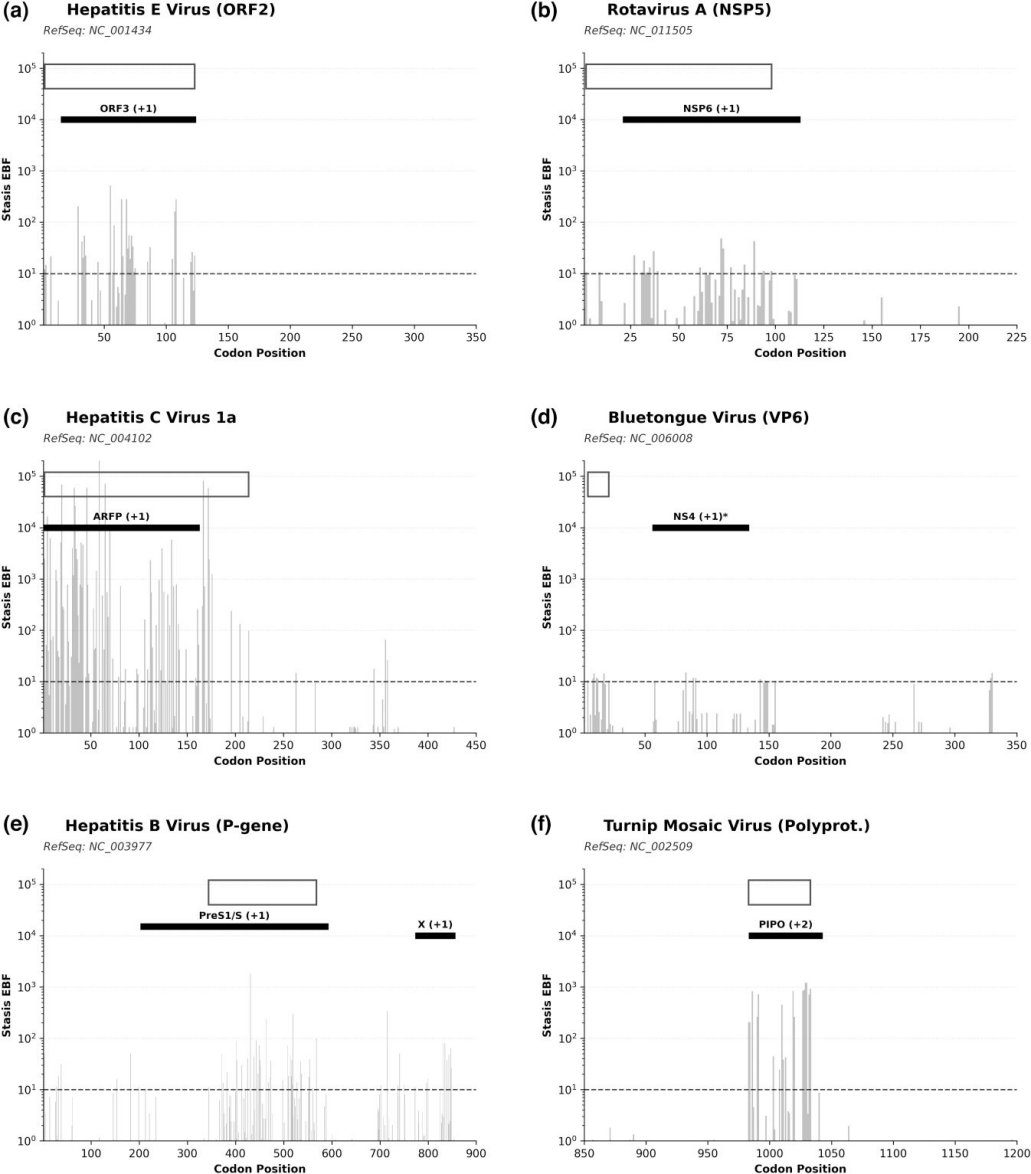
Mapping of B-STILL Stasis Clusters to annotated viral gene overlaps. Horizontal tracks indicate RefSeq overlaps (black lines) and statistically significant Stasis Clusters (dark gray rectangles).

To evaluate whether the spans of regional Stasis Clusters correspond to genome regions that are expected to evolve under severe constraints, we applied the Hypergeometric Scan Statistic to the 110-gene FRESCO viral dataset (Sealfon et al. 2015), where viral genomes were analyzed for the presence of overlapping reading frames. We utilized a significance threshold of EBF≥10 to define the proximal ESAs, a choice supported by simulation results that demonstrate that this threshold maintains a Null FPR ≪1%. Application of the scan statistic identified 45 statistically significant regional Stasis Clusters (Table S3). B-STILL regional clusters mapped to documented overlapping reading frames across diverse viral families (Fig. 5). In hepatitis E virus ORF2 (Fig. 5a), B-STILL identified a Stasis Cluster spanning 122 codons (sites 2–123, $p=2.7\times 10^{-16}$) that encompasses the 110-codon overlap with ORF3 (sites 15–124). Similar clusters were identified for the protein F frameshift in hepatitis C (HCV1a sites 2–214, $p=4.4\times 10^{-20}$; Fig. 5c), the NSP6 overlaps in rotavirus A (NSP5 sites 2–98, $p=1.5\times 10^{-7}$; Fig. 5b), and the PIPO overlaps in turnip mosaic virus (polyprotein sites 983–1,033, $p=1.7\times 10^{-14}$; Fig. 5f).

However, detection of overlapping reading frames is not exhaustive. Of the 110 viral genes analyzed, several annotated overlaps remained undetected, particularly in cases where the primary or secondary reading frames exhibited low background substitution opportunity or relatively relaxed selection. This suggests that B-STILL targets the subset of features under significant purifying selection, in the context of a sufficiently variable genomic background. Furthermore, we identified numerous Stasis Clusters that do not correspond to annotated overlapping open reading frames (ORFs) (Table S3). These “unmapped” regional Stasis Cluster signals likely correspond to other multilayered functional elements, such as structured *cis*-regulatory RNA motifs, viral packaging signals, or biologically relevant nucleic acid secondary structural elements.

### Stasis Clusters in Mammals and the Dark Proteome

**Fig. 6.**
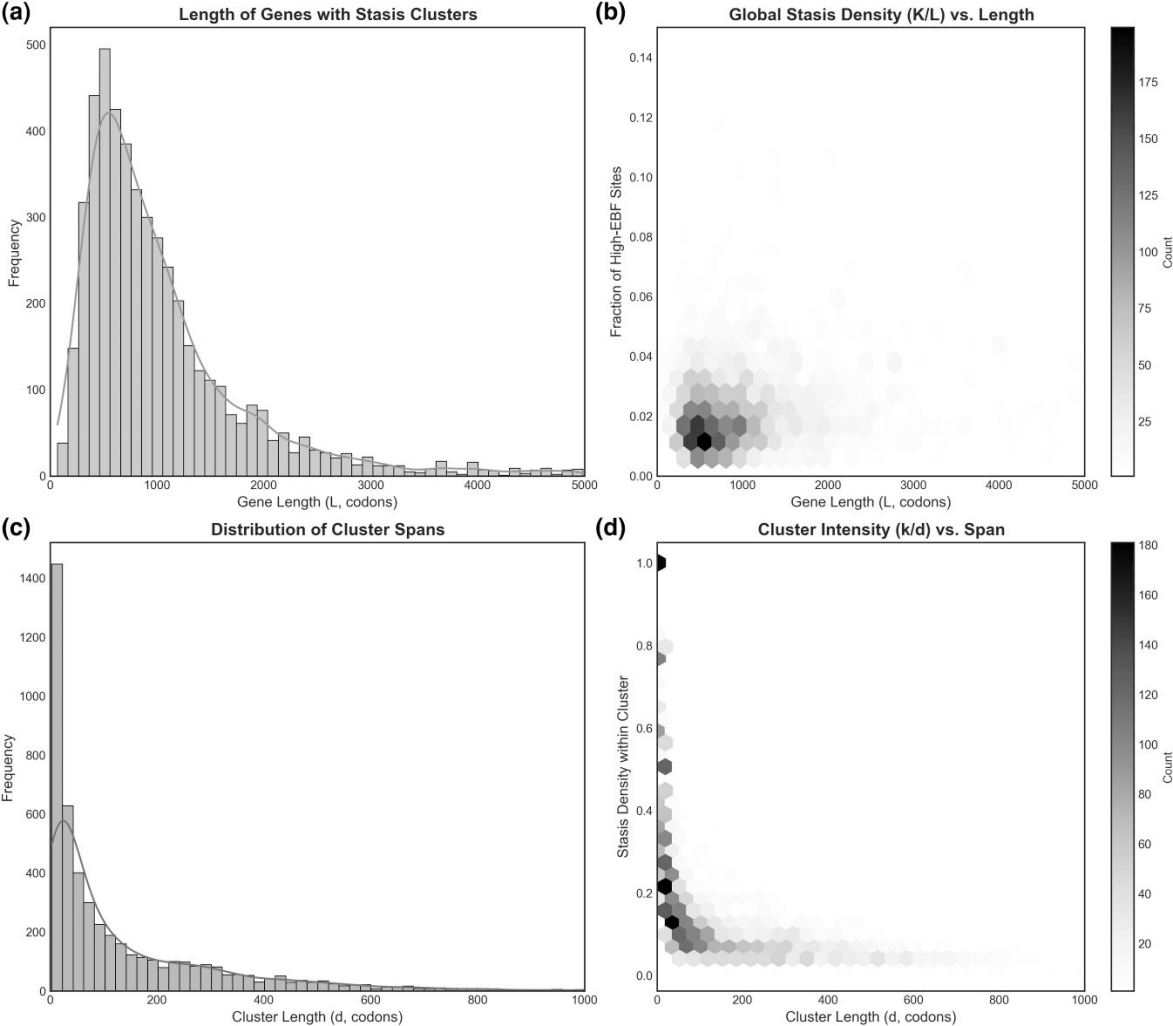
Statistical characterization of 4,888 evolutionary Stasis Clusters identified across 19,152 mammalian genes according to gene length (in 100 codon bins; a and b) or length of the Stasis Clusters (in 20 codon bins; c and d).

The global scan of the mammalian exome identified 4,888 statistically significant Stasis Clusters across 19,152 genes (Fig. 6). The identified clusters exhibit a median span of 52 codons (Fig. 6c and d) and are nonuniformly distributed across the exome (Fig. 6a and b), with ESA density peaking in genes involved in core cellular processes. Formal enrichment analysis using Fisher’s exact test against the InterPro database (Blum et al. 2025) identified 133 functional associations (Table S4). We identified a significant enrichment of Stasis Clusters within genes associated with RNA-binding (OR=2.4, $p<10^{-15}$), DNA-templated transcription (OR=1.8, $p<10^{-12}$), and structural stabilization of the cytoskeleton (OR=1.6, $p<10^{-8}$). Individual Stasis Clusters are nonrandomly localized within critical protein architectures, including mapping to the DNA-binding domain in TP53, the Armadillo repeat regions in APC and NF1, and numerous zinc finger motifs. These results demonstrate that B-STILL identifies regional selective footprints that correspond to the functional regions of the proteome.

Application of the B-STILL framework to 815 uncharacterized protein-coding genes (the “dark proteome”) of the 120-species mammalian alignment (Hecker and Hiller 2020) identified 1,342 ESAs and 8 statistically significant regional Stasis Clusters (Fig. S3). In these proteins of unknown function, the discovery of contiguous ESAs provides a data-driven approach to identifying functional modules.

One such example is found in FAM214A, which contains a 239-codon Stasis Cluster ($p=1.4\times 10^{-18}$, sites 822–1,060) harboring multiple ESAs (EBF>500). Statistical analysis of the FAM214A structural model reveals that these signals are statistically significantly clustered in three-dimensional space ($p<10^{-4}$, permutation test; Fig. S4). We identified a structural hub centered at residue 1,038, which contains nine ESAs within a 10 Å radius. Similarly, we identified a 1,812-codon cluster in C2ORF16 ($p=8.3\times 10^{-14}$) and a small 3-residue Stasis Cluster in C1ORF167 ($p=6.9\times 10^{-7}$, sites 209–211). These results demonstrate that regional Stasis Clusters can pinpoint the structural frameworks or interaction interfaces of uncharacterized proteins, providing targets for experimental functional characterization.

### Extremes of the Mammalian Evolutionary Stasis Landscape

We identified a subset of genes harboring long contiguous Stasis Clusters spanning more than 1,000 codons. The longest cluster is found at the C-terminus of MUC16, where a 2,063-codon cluster ($p=1.8\times 10^{-24}$) corresponds to a series of 16 tandemly repeated SEA domains. While alignment inaccuracies typically increase apparent variation, B-STILL identifies contiguous blocks of zero-variation, suggesting that the stasis signal is a biological property of these regions. Similar long Stasis Clusters were identified in NEB (Nebulin, 1,193 codons), where absolute sequence constraint maintains the precise molecular spacing required for muscle thin-filament regulation. Conversely, the high-intensity tail of the distribution identifies “functional snaps” (short, localized regions of absolute sequence conservation, akin to a “cold snap”): short, contiguous intervals of absolute or near-absolute sequence stasis (k/d≥0.80) where nearly every codon is an ESA. These motifs frequently map to critical catalytic or binding interfaces (Table S4). For instance, we identified a six-residue snap in KPNB1 (Importin *β*-1, sites 38–43, $p=1.6\times 10^{-8}$) mapping to a structural ARM repeat, and a seven-residue snap in SIM2 mapping to a functional PAC motif ($p=4.8\times 10^{-12}$). Other notable snaps include a six-residue Stasis Cluster in the Rab GTPase-activating domain of EVI5L ($p=7.5\times 10^{-14}$) and a three-residue cluster in the RNA polymerase II clamp domain (POLR2A, sites 11–13).

### Comparative Resolution of B-STILL vs. PhyloP

**Fig. 7.**
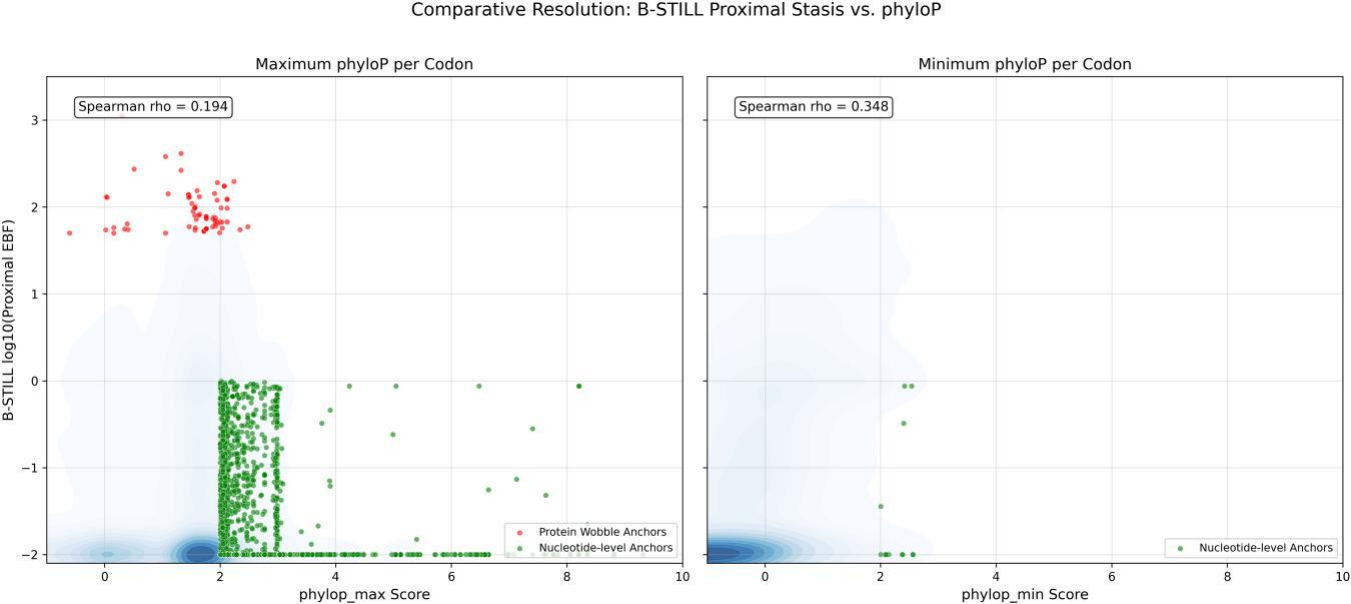
Comparative resolution of B-STILL proximal stasis EBF vs. nucleotide-level conservation (phyloP) across 38 mammalian genes. (Left) Correlation with the maximum phyloP score per codon (ρ=0.19). The visible “0/0 plateau” represents the resolution ceiling of frequentist LRTs, which assign an identical significance score to all invariant sites, failing to distinguish between stochastic invariance and functional constraint. (Right) Correlation with the minimum phyloP score per codon (ρ=0.35). The stronger alignment with the least-conserved position indicates that B-STILL EBFs effectively filter out genetic-code artifacts—where positions are invariant solely due to codon-structure constraints—identifying functional anchors with higher specificity.

Head-to-head benchmarking against phyloP across 38 representative mammalian genes demonstrates that B-STILL provides higher resolution for functional ESAs than standard nucleotide-based LRTs (Fig. 7). While there is a general correlation between metrics, the relationship is stronger when comparing B-STILL Proximal EBFs to the minimum (worst) phyloP score within a codon (ρ=0.35) rather than the maximum (ρ=0.19). This result highlights a distinction in how these models operate: because standard nucleotide-based methods treat sites independently, they often assign high significance to first or second codon positions that are invariant due to intense purifying selection on the encoded amino acid. This creates a “0/0 plateau” where frequentist scores saturate at a significance ceiling; because the LRT is a function of the observed data, all invariant sites across a phylogeny receive an identical score regardless of their underlying mutational opportunity. B-STILL, by modeling the codon as the fundamental unit of selection and leveraging a hierarchical prior, breaks this resolution ceiling by ranking stasis according to its statistical surprise relative to the gene-wide background.

This distinction is illustrated by two emergent archetypes of conservation. The first, termed “protein wobble anchors,” consists of sites where B-STILL identifies stasis (proximal EBF>100) while the maximum phyloP score remains low (<3.0). At site 59 of APOBEC3D, for instance, B-STILL reports a proximal EBF of 1,124.3, while the maximum phyloP score is 0.30. Similarly, at site 2,103 of F8 (proximal EBF=272.8, max phyloP=0.51) and site 2,777 of BRCA2 (proximal EBF=381.0, max phyloP=1.05), B-STILL identifies purifying selection that is not captured by nucleotide-level tools. In these cases, the protein sequence is invariant across 120 mammals, but the underlying codons exhibit synonymous variation (eg GTA vs. GTG at APOBEC3D site 59). Standard nucleotide-based methods conflate this synonymous variation with a lack of selective constraint, resulting in lower scores. B-STILL identifies that the absence of amino-acid substitutions in the face of background synonymous opportunity is a signature of functional constraint.

Conversely, the comparison identifies “nucleotide-level anchors,” where phyloP scores are high but B-STILL Proximal EBFs remain low. These sites often correspond to positions where both synonymous and nonsynonymous substitutions are rare at the nucleotide level, but B-STILL identifies amino-acid variation that suggests the position is not evolutionarily invariant at the protein level. For instance, at site 5 of TP53, phyloP yields a score of 2.68, whereas B-STILL assigns a negligible proximal EBF ($7.6\times 10^{-8}$). Alignment analysis reveals that while the consensus CAG (glutamine) is prevalent, multiple lineages exhibit nonsynonymous transitions to proline (CCG) and leucine (CTG), identifying that the site is not a strict functional anchor.

### Computational Performance

Benchmarks across 19,152 genes demonstrate the high-throughput performance of B-STILL (Table S5), completing the analysis of nearly 30 million codons in approximately 422 CPU-hours on a cluster of Ampere Altra (3.0 GHz) ARM64 compute nodes. The framework achieved a mean throughput of 19.52 codons per second/per core, establishing it as a scalable tool for high-resolution genomic mapping.

## Discussion

B-STILL provides a principled Bayesian framework for quantifying selective and evolutionary constraint. A central conceptual advancement of this work is the surmounting of the “0/0 plateau” that limits the standard frequentist tools that are commonly used to quantify conservation and purifying selection. Because LRTs are functions of the observed data, they exhibit a resolution ceiling where all invariant sites in a given phylogeny receive an identical maximal score. By conditioning the stasis signal on site-specific synonymous evolutionary opportunity and gene-wide selective priors, B-STILL breaks this degeneracy, providing a ranking of evolutionary stasis based on statistical surprise. An example of this are “Protein Wobble Anchors”—sites where extreme evolutionary stasis persists despite high synonymous substitution opportunity. This hierarchical design effectively filters out the global selective background: while an invariant site in an ultra-conserved gene (eg a histone) is expected and thus yields a moderate EBF, a single invariant anchor in a rapidly diversifying gene (eg an olfactory receptor) represents a substantial deviation from the neutral expectation and is prioritized accordingly. This ensures that B-STILL identifies the highly constrained regions of a protein relative to its specific evolutionary context, rather than merely re-stating global conservation.

A key operational parameter in B-STILL is the stasis radius *X* (the expected substitution threshold). Because the stasis regime is defined in terms of expected substitutions (E[S]≤X) rather than rate parameters directly, the rate boundary scales dynamically based on the evolutionary depth (tree length *T*) of the alignment: $(\alpha \cdot d_{s}+\beta \cdot d_{\mathrm{ns}})\leq X/T$. In deep trees (large *T*), the stasis boundary in rate space is tight, ensuring that only sites with extremely low rates are classified in stasis. In shallow trees (small *T*), the rate boundary expands, but because the neutral prior expectation of zero substitutions is high, the EBF is compressed toward 1.0, automatically preventing false-positive constraint calls. The default threshold of X=0.5 balances sensitivity and conservatism, representing the point where the expected number of substitutions across the tree is small enough that seeing zero changes is indicative of potential stasis under Poisson expectations. However, users can tune this boundary depending on the divergence and quality of their data. In datasets with deep trees where sequencing errors or rare, selectively neutral “leaky” substitutions are expected, increasing *X* to 1.0 can prevent false-negative calls. Conversely, in highly conserved alignments where strict sequence immutability is required to infer constraint, decreasing *X* to 0.1 ensures that only sites under absolute purifying selection are detected.

The framework’s codon-aware design provides an advantage for identifying regulatory stasis that persists at the nucleotide level independently of amino-acid change. Our validation against the ClinVar database demonstrates that Synonymous Stasis (${\text{EBF}}_{\mathrm{syn}}$) is a predictor of protein sites at which any amino-acid substitutions are likely to be clinically pathological (aggregate AUROC=0.88); identifying critical regulatory sites in genes like TP53 and CFTR that are not resolved by ensemble protein-focused predictors of clinical relevance such as REVEL. Interestingly, B-STILL’s predictive power for synonymous variants (AUROC=0.88) exceeds its performance for missense variants (AUROC=0.65). This difference in performance likely reflects the fact that the pathogenicity of synonymous substitutions is driven by relatively pure nucleotide-level constraints (eg splicing enhancers, mRNA stability) that are well-captured by a phylogenetic baseline. In contrast, missense pathogenicity is a composite of phylogenetic, structural, and biochemical features; by providing a highly specific phylogenetic prior, B-STILL complements rather than replaces modern ensemble methods, which often struggle to calibrate the “evolutionary floor” in the absence of protein-sequence change.

The integration of site-level ESAs into regional Stasis Clusters revealed larger-scale regions of extreme evolutionary constraint across diverse genomic architectures. In viral genomes, B-STILL delineates overlapping reading frames with high precision, identifying the intervals where multilayered coding requirements enforce sequence conservation. In the mammalian exome, these regional Stasis Clusters map to critical architectural features, including the SEA domain repeats in MUC16, the precise molecular spacing interfaces in NEB, and structural motifs such as the ARM repeats in KPNB1. The discovery of these clusters independently of structural or biochemical data suggests that regional stasis is a robust proxy to identify the highly constrained functional modules of the proteome.

B-STILL is particularly suited for functional annotation within the “dark proteome.” In uncharacterized ORFs where biochemical data are absent, the spatial clustering of ESAs provides a data-driven strategy for identifying critical residues. The discovery of a statistically significant structural hub in FAM214A ($p<10^{-4}$) provides a prioritized target for experimental characterization, demonstrating that distribution across a gene of ESAs can demarcate potentially functional protein interfaces in the absence of any known homology or mechanistic biochemical data.

Finally, while the genome annotation field is increasingly moving toward phylogeny-aware gLMs such as GPN-Star and AlphaGenome, B-STILL remains a tool for high-resolution, mechanistic-agnostic annotation. Unlike deep learning models that synthesize vast contextual windows through complex neural architectures, B-STILL provides a transparent, data-driven codon-level map of selective constraint derived directly from the underlying substitution process. By focusing on the genomic syntax of coding regions across intermediate to deep evolutionary timescales, B-STILL provides principled codon-level estimates of functionally relevant evolutionary stasis that complement, and could therefore also productively augment, modern AI-driven genome annotation tools.

## Supplementary Material

evag184_Supplementary_Data

## Data Availability

B-STILL is implemented in HyPhy (v2.5.95+). Source code, analysis scripts, and simulation datasets are available under the MIT license at https://github.com/veg/hyphy and https://github.com/veg/b-still.
