# Supplementary material for: Beyond Invariable Sites: Using Evolutionary Stasis to Map Multilayered Constraints on the Evolution of Viral and Mammalian Genomes
Source: Genome Biol Evol. 2026 Jul 28;18(8):evag184. doi: 10.1093/gbe/evag184 (PMC13427764; doi:10.1093/gbe/evag184)
Supplement: evag184_Supplementary_Data [file evag184_supplementary_data.zip › Supplementary Material.pdf]

Supplementary Material

S1 Methodological Dissections

S1.1 phyloP: Phylogenetic P-values

The *phyloP* tool, part of the PHAST suite, represents perhaps the most direct application of likelihood ratio tests (LRT) to site-level conservation (Pollard et al., 2010).

- Estimation Logic: For each site, *phyloP* evaluates the observed number of substitutions against a pre-calibrated neutral phylogenetic tree. The null hypothesis assumes that substitutions follow a neutral Markov process (typically calibrated using four-fold degenerate sites).
- Quantification of Invariance: For a strictly invariant site, the surprisingness is a direct function of the total neutral tree length (T). Under a Poisson approximation, the probability of zero substitutions is e^{-T}. *phyloP* outputs a score defined as $-\log_{10}(p\text{-value})$, where p is the probability of the data under the neutral model.
- Limitation: As a site-independent method, it lacks the ability to share information across the gene, making it highly sensitive to the accuracy of the provided neutral tree.

S1.2 GERP++: Genomic Evolutionary Rate Profiling

GERP++ eschews p-values in favor of a count-based metric known as Rejected Substitutions (RS) (Davydov et al., 2010).

- Estimation Logic: The method calculates the expected number of substitutions (E) that would have occurred at a site under neutral evolution given the tree topology and branch lengths. It then subtracts the observed number of substitutions (O).
- Quantification of Invariance: For an invariant site ($O = 0$), the RS score is exactly equal to the neutral tree length ($RS = E$). This provides an intuitive, additive measure of constraint: an RS score of 5.0 implies that evolution has successfully "rejected" five expected mutations.
- Limitation: Like *phyloP*, *GERP++* treats sites in isolation, failing to account for the gene-wide distribution of selective pressures.

S1.3 phastCons: Spatial Context via HMMs

Unlike independent site methods, *phastCons* utilizes a Phylogenetic Hidden Markov Model (phylo-HMM) to contextualize conservation (Siepel et al., 2005).

- Estimation Logic: The model toggles between two hidden states: "neutral" and "conserved." The state transitions are governed by a set of parameters that reflect the expected length of conserved elements.
- Quantification of Invariance: It assigns a posterior probability (from 0 to 1) that a site belongs to the conserved state. A strictly invariant site flanked by high variation will receive a lower probability than one residing within a cluster of conserved positions.
- Limitation: While effective for discovering conserved motifs, the smoothing effect of the HMM can obscure sharp, site-specific signatures of purifying selection.

S1.4 Rate4Site: Empirical Bayesian Rate Inference

For amino acid alignments, *Rate4Site* is the established gold standard for quantifying protein-level conservation (Pupko et al., 2002).

- Estimation Logic: The method uses Empirical Bayesian inference to assign a relative evolutionary rate to every site, incorporating a substitution matrix (e.g., WAG or JTT) and the phylogeny.
- Quantification of Invariance: An invariant site yields a posterior probability distribution sharply peaked at the lowest possible rate. This allows for a continuous ranking of sites even when multiple sites appear identical at the sequence level.

- Limitation: It typically assumes a single global rate for the entire protein, which is fundamentally insufficient for capturing the dN/dS dynamics essential for codon-level analysis.

S1.5 Mechanistic Codon Models (HyPhy / PAML)

Codon-based models provide a more nuanced view of selection by estimating the ratio of non-synonymous to synonymous substitutions ($\omega = \beta/\alpha$) (Kosakovsky Pond and Frost, 2005; Yang, 2007).

- Estimation Logic: These models use synonymous drift (α) as an internal control. Conservation is inferred when $\beta < \alpha$.
- Quantification of Invariance: Methods like FEL or FUBAR estimate the posterior probability $P(\beta < \alpha \mid \text{Data})$. However, at strictly invariant sites where $\alpha = \beta = 0$, the standard selection test is often statistically uninformative.
- Conceptual Gap: This methodological failure at the limit of zero substitutions is the primary motivation for the high-resolution grid and EBF approach implemented in B-STILL.

S1.6 EVE: Generative Deep Learning

The EVE (Evolutionary model of Variant Effect) method utilizes Variational Autoencoders (VAEs) trained on deep sequence alignments (Frazer et al., 2021).

- Estimation Logic: EVE learns the latent multi-dimensional distribution of sequences. It quantifies conservation through the "unlikeliness" of a mutation within the learned manifold.
- Quantification of Invariance: For invariant sites, the model's latent space dictates that any amino acid other than the consensus has an extremely low probability.
- Limitation: While powerful, these models require large-scale alignments and are often computationally prohibitive compared to grid-based Bayesian methods.

S2 Standardizing the Neutral Baseline for Benchmarking

To rigorously compare B-STILL against site-independent tools like phyloP and GERP++, it is essential to establish a conceptually equivalent neutral baseline. Traditionally, these tools rely on neutral trees calibrated using four-fold degenerate sites—an approximation that is fundamentally insufficient as it discards the majority of the synonymous signal and introduces potential compositional biases.

We propose a Codon-derived Neutrality protocol for benchmarking:

1. Synonymous Tree Estimation: Utilize a mechanistic codon model (e.g., MG94) to estimate the synonymous substitution rate (α) across the entire alignment. This leverages every synonymous substitution opportunity, yielding a significantly more stable and data-dense phylogeny than 4-fold site counting.
2. Neutral Reference Extraction: The true neutral baseline for a coding region is defined as the tree where branch lengths (L) represent expected synonymous change ($L_{neutral} = \alpha \times T$). This tree represents the expected drift at a site where $\beta = \alpha$.
3. Cross-Tool Calibration: Export this synonymous tree and the associated GTR substitution parameters into a PHAST-compatible `.mod` file. This ensures that the "surprise" measured by phyloP is statistically comparable to the constraint inferred by B-STILL, moving the comparison from one of data-proxies to one of statistical inference frameworks.

S3 Supplementary Tables and Figures

Site	α	β	P[prox]	EBF[prox]	Codon Composition
4	0.701	0.138	0.6586	12.47	CCT (162), CCC (4), YCT (1), ...
5	0.357	0.048	0.8946	54.89	ATT (167), RTT (1)
7	0.287	0.040	0.9338	91.18	ACT (183), RCT (1)
10	0.676	0.039	0.7715	21.83	GTA (182), GTG (2)
14	0.244	0.036	0.9540	134.08	CCA (184)
17	0.515	0.039	0.8221	29.87	GAT (200)
25	0.590	0.127	0.7520	19.61	CCA (200), GCA (2), CCR (1), ...
27	0.657	0.034	0.7896	24.26	ACA (201), ACG (2), WCA (1), ...
30	0.828	0.041	0.6708	13.17	AAA (201), AAR (3), AAG (1)
31	0.922	0.030	0.6422	11.60	ATA (204), WTA (2)
33	0.233	0.036	0.9580	147.49	GCA (206)
51	0.765	0.017	0.8497	36.53	GGG (460), GGA (14), GGT (2)
52	0.671	0.017	0.8269	30.89	CCT (472), CCC (2), CCW (2)
59	0.775	0.017	0.7660	21.16	CCA (469), CCG (4), CCR (3)
105	0.579	0.025	0.8871	50.81	TCA (471), TCG (3), TCR (2)
113	0.729	0.020	0.7516	19.56	GAT (465), RAT (6), GAC (2), ...
117	0.102	0.028	0.9968	1990.84	TCA (474), TCR (1), TMA (1)
129	0.731	0.071	0.7673	21.32	GCA (464), GCC (4), GCT (3), ...
137	0.726	0.021	0.7524	19.65	AAT (471), AAC (2), ART (1), ...
140	0.586	0.018	0.8863	50.39	CCA (470), CCM (2), CCC (2), ...
156	0.923	0.022	0.6183	10.47	TCA (465), TCR (4), TCG (4), ...
185	0.270	0.022	0.9470	115.56	GAT (476)
189	0.096	0.158	0.9796	310.99	GTA (470), ATA (2), GTR (2), ...
209	0.945	0.031	0.6617	12.64	CTG (387), CTA (37), TTG (25), ...
217	0.106	0.020	0.9968	1986.41	CCA (464), CCW (5), CCM (1)
222	0.845	0.034	0.7362	18.04	CAG (453), CAA (13), CAR (3)
232	0.274	0.030	0.9432	107.39	TAT (467), TAY (2)
240	0.934	0.018	0.6106	10.14	ACA (453), ACG (4), ACR (2), ...
247	0.636	0.019	0.8519	37.19	CCA (428), CCT (2), SCA (1), ...
254	0.514	0.052	0.8658	41.72	GTC (146), GTT (2)
255	0.627	0.051	0.7587	20.33	AAT (148)
290	0.903	0.046	0.6222	10.65	ACA (117), ACG (2)
299	0.447	0.061	0.8379	33.42	GCA (89), GSA (2), GCR (2)
300	0.707	0.071	0.7107	15.88	GAG (86), GAA (5), GRG (1)
310	0.454	0.219	0.6793	13.70	CTA (35), YTA (1)

Supplementary Table S1: Sites in HIV-1 RT inferred to be under significant proximal constraint (EBF $\geq$ 10). α and β represent the mean posterior synonymous and non-synonymous rates, respectively.

Gene	Scenario	Mean Sig	Std Dev	Max Sig
ENCenv	A	0.46	0.95	4
ENCenv	B	1.32	1.45	7
ENCenv	C	0.00	0.00	0
HIV_RT	A	0.60	0.90	5
HIV_RT	B	1.36	1.10	5
HIV_RT	C	3.56	2.26	10
SARS-CoV-2-spike	A	0.00	0.00	0
SARS-CoV-2-spike	B	0.00	0.00	0
SARS-CoV-2-spike	C	0.00	0.00	0
bglobin	A	0.30	0.51	2
bglobin	B	0.02	0.14	1
bglobin	C	0.12	0.33	1
camelid	A	0.00	0.00	0
camelid	B	0.06	0.24	1
camelid	C	0.00	0.00	0
rbcL	A	0.00	0.00	0
rbcL	B	0.12	0.33	1
rbcL	C	0.48	0.68	2

Supplementary Table S2: Summary of B-STILL performance across 900 simulation replicates (50 per Gene/Scenario combination). Scenario A represents the neutral null model; Scenario B evaluates extreme purifying selection ($\omega = 0.1$) at all sites; Scenario C tests sensitivity under reduced evolutionary depth (0.5$\times$ tree scaling).

Virus	Gene (L)	Cluster (Codons)	k/d	P-value	Overlapping ORF (Range)
CCHV	glycoprotein	770–783	4/14	1.9×10^{-06}	None
DENV1	DENV1	12–955	15/944	6.9×10^{-18}	None
DENV2	DENV2	13–811	24/799	1.1×10^{-14}	None
EBOV	all_genes	3298–3308	4/11	1.5×10^{-06}	None
FMDV	ns	1092–1377	21/286	7.6×10^{-13}	None
FMDV	polyprotein	1024–1184	22/161	2.1×10^{-09}	None
FMDV	polyprotein	2087–2372	44/286	8.7×10^{-21}	None
HBV	HBV P-gene	344–568	34/225	3.8×10^{-08}	large envelope protein (202–593)
HCV1a	HCV 1a Polyprot.	2–214	81/213	4.4×10^{-20}	protein F (1–163)
HCV1a	HCV 1a Polyprot.	2475–3037	153/563	3.6×10^{-15}	None
HCV1b	HCV 1b Polyprot.	2–213	92/212	3.6×10^{-21}	protein F (1–162)
HCV1b	HCV 1b Polyprot.	2491–2608	27/118	3.2×10^{-07}	None
HCV1b	HCV 1b Polyprot.	2684–3037	97/354	7.0×10^{-15}	None
HepA	polyprotein	17–27	4/11	5.6×10^{-07}	None
HepA	polyprotein	1749–1809	6/61	1.8×10^{-08}	None
HepE	ORF1	3–32	15/30	8.6×10^{-25}	None
HepE	HepE ORF2	2–123	31/122	2.7×10^{-16}	hypothetical protein (15–124)
HepE	HepE ORF2	402–406	4/5	4.1×10^{-05}	None
HepE	ORF3	71–91	6/21	1.3×10^{-04}	None
JEV	polyprotein	800–1349	17/550	1.5×10^{-13}	None
Newcastle	P	133–287	8/155	1.4×10^{-05}	None
PVY	PVY Polyprot.	2478–3066	26/589	2.7×10^{-12}	None
TuMV	TuMV Polyprot.	983–1033	18/51	1.7×10^{-14}	PIPO (983–1043)
TuMV	TuMV Polyprot.	3004–3166	77/163	3.4×10^{-30}	None
VEEV	structural	815–1242	13/428	3.6×10^{-10}	None
WNV	wnv	2–1173	30/1172	3.4×10^{-24}	None
bluetongue	NS2	344–351	4/8	1.1×10^{-07}	None
bluetongue	NS3	66–81	6/16	7.7×10^{-06}	None
bluetongue	VP1	2–15	6/14	3.4×10^{-09}	None
bluetongue	VP1	1299–1302	3/4	6.1×10^{-06}	None
bluetongue	VP3	889–900	4/12	5.7×10^{-06}	None
bluetongue	VP4	2–14	5/13	6.1×10^{-07}	None
bluetongue	BTV VP6	4–21	8/18	2.6×10^{-07}	None
enterovirusa71	enterovirusa71	1224–1233	6/10	3.8×10^{-14}	None
poliovirus	ns	361–370	8/10	2.3×10^{-14}	None
poliovirus	ns	1157–1221	10/65	4.5×10^{-10}	None
poliovirus	poliovirus	1275–2238	25/964	7.5×10^{-16}	None
rotavirus	NSP2	2–31	7/30	4.9×10^{-09}	None
rotavirus	NSP3	2–9	3/8	1.1×10^{-05}	None
rotavirus	NSP4	155–175	3/21	1.5×10^{-03}	None
rotavirus	Rotavirus NSP5	2–98	20/97	1.5×10^{-07}	NSP6 (21–113)
rotavirus	VP1	2–29	7/28	3.7×10^{-11}	None
rotavirus	VP2	2–7	3/6	1.3×10^{-05}	None
rotavirus	VP3	2–223	12/222	3.8×10^{-17}	None
rotavirus	VP7	5–35	5/31	1.6×10^{-06}	None

Supplementary Table S3: Statistically significant Stasis Clusters identified by Hypergeometric scan statistic (10,000 permutations) on FRESCO viral alignments. k represents stasis sites ($EBF \geq 10$) in cluster span d.

Functional Feature	Type	Cluster Count
Armadillo	Repeat	6
Armadillo/beta-catenin-like repeat	Repeat	3
Nucleic acid-binding proteins	Homologous Superfamily	3
Nucleic acid-binding, OB-fold	Homologous Superfamily	3
Microtubule associated protein, tubulin-binding repeat	Repeat	3
Tau and MAP protein, tubulin-binding repeat	Family	3
Tau and MAP proteins tubulin-binding repeat profile	Domain	3
Armadillo-type fold	Homologous Superfamily	2
Vacuolar membrane-associated protein Iml1	Family	2
Vacuolar membrane-associated protein Iml1, N-terminal domain	Domain	2
DEP DOMAIN CONTAINING PROTEIN 5	Family	2
Tau and MAP proteins tubulin-binding repeat signature	Conserved Site	2
Methyl-CpG binding protein MeCP2	Family	2
SEA domain	Domain	2
P53 DNA-binding domain	Domain	2
Endoplasmic Reticulum Stress-Regulated Transcription Factor	Family	2
Helix hairpin bin	Homologous Superfamily	1
Leucine-rich Repeat Variant	Homologous Superfamily	1
Armadillo-like helical	Homologous Superfamily	1
Adenomatous polyposis coli (APC) family	Family	1

Supplementary Table S4: Top 20 functional protein features significantly enriched for B-STILL Stasis Clusters across the mammalian exome. Enrichment scores were calculated relative to the genomic background via Fisher's Exact Test; all listed features exhibit $p < 10^{-50}$ after multiple testing correction.

Metric	Value
Total genes analyzed	19,152
Total codons analyzed	29,691,139
Mean overall runtime per gene	79.4 s
Median overall runtime per gene	74.0 s
Mean throughput	19.52 codons/s
Total CPU-hours (Ampere Altra 3.0 GHz)	$\approx$ 422 h

Supplementary Table S5: Computational performance of the B-STILL framework across the 120-species mammalian exome dataset. Benchmarks were recorded on a cluster of Ampere Altra (3.0 GHz) ARM64 compute nodes.

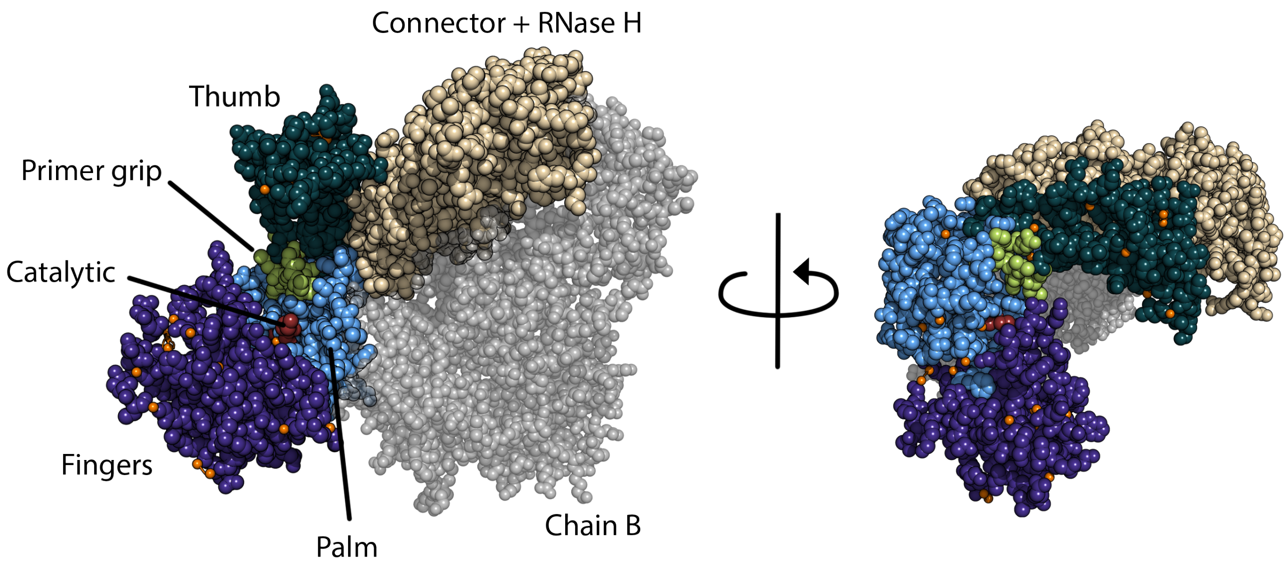

Figure S1: Structural mapping of invariant sites in HIV-1 Reverse Transcriptase (PDB: 1RTD) with a global view of B-STILL inferred invariant residues (red spheres) projected onto the RT heterodimer.

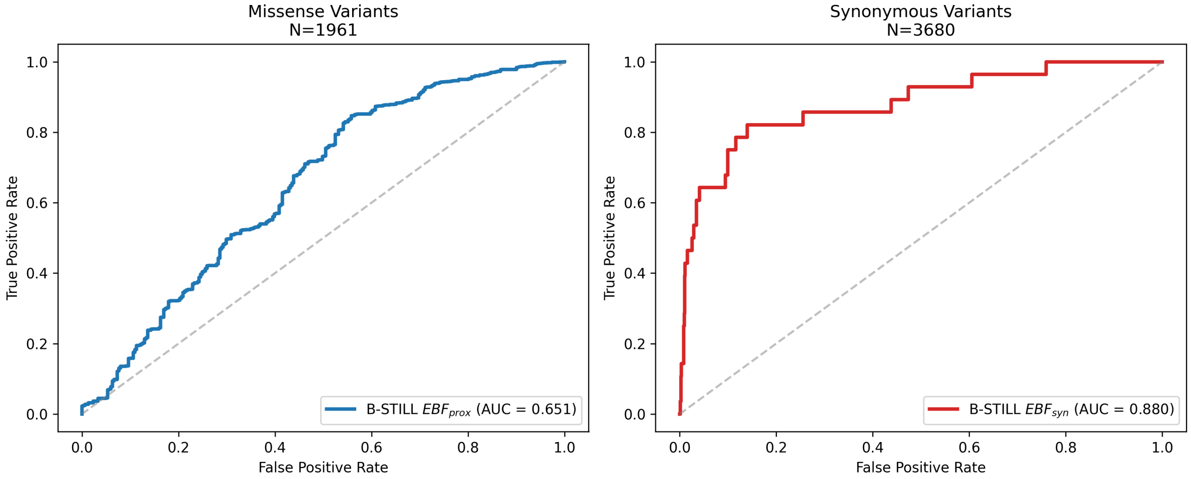

Figure S2: Clinical validation of B-STILL Empirical Bayes Factors using pathogenic and benign variants from ClinVar. (Left) Aggregate ROC curve for non-synonymous variants (AUROC = 0.65), using EBF_{prox} as the predictor. (Right) Aggregate ROC curve for synonymous variants (AUROC = 0.88) using EBF_{syn}. The True Positive Rate (TPR) represents the proportion of clinically confirmed pathogenic variants correctly identified as Evolutionary Stasis Anchors, while the False Positive Rate (FPR) denotes the proportion of confirmed benign variants incorrectly flagged.

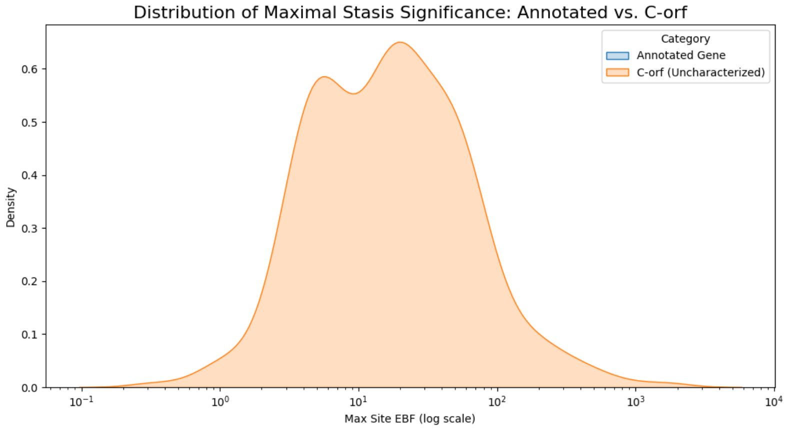

Figure S3: Distribution of Evolutionary Stasis Anchor significance (EBF) across 815 uncharacterized mammalian ORFs. The heavy-tailed distribution highlights a subset of sites under extreme constraint in the dark proteome.

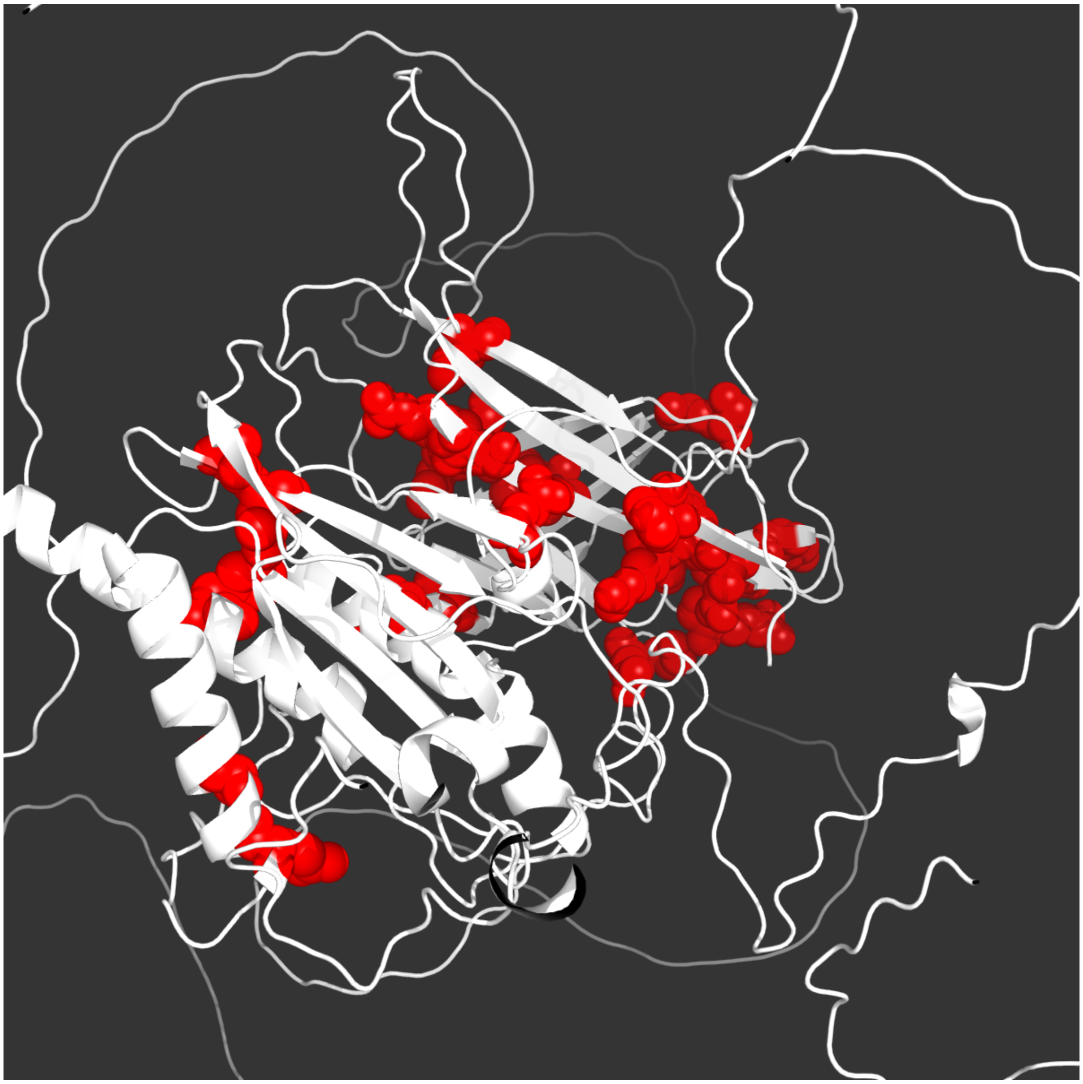

Figure S4: Three-dimensional structural hub of Evolutionary Stasis Anchors in the uncharacterized protein FAM214A. Residues are colored by B-STILL significance ($\log_{10}$ EBF), with significant anchors (EBF $\geq$ 100) shown as red spheres. We identified a significant Stasis Cluster comprising nine Evolutionary Stasis Anchors centered at residue 1038 ($p < 10^{-4}$, permutation test), likely demarcating a structural or interaction hub in this poorly characterized ORF.
