# Supplementary material for: Beyond Invariable Sites: Using Evolutionary Stasis to Map Multilayered Constraints on the Evolution of Viral and Mammalian Genomes
Source: Genome Biol Evol. 2026 Jul 28;18(8):evag184. doi: 10.1093/gbe/evag184 (PMC13427764; doi:10.1093/gbe/evag184)
Supplement: evag184_Supplementary_Data [file evag184_supplementary_data.zip › Supplementary References.docx]

References:

Frazer, J., Notin, P., Dias, A., Gomez, A., Min, J. K., Brock, K., Gal, Y., and Marks, D. S. 2021. Disease variant prediction with deep generative models of evolutionary data. Nature, 599(7883): 91–95.

Pupko, T., Bell, R., Mayrose, I., Glaser, F., and Ben-Tal, N. 2002. Rate4site: an algorithmic tool for the identification of functional regions in proteins by evolutionary rate of amino acid sites. Bioinformatics, 18(suppl 1): S71–S77.
